# Local and global predictors of synapse elimination during motor learning

**DOI:** 10.1126/sciadv.adk0540

**Published:** 2024-03-15

**Authors:** Nathan G. Hedrick, William J. Wright, Takaki Komiyama

**Affiliations:** ^1^ Department of Neurobiology, University of California San Diego, La Jolla, CA, USA.; ^2^ Center for Neural Circuits and Behavior, University of California San Diego, La Jolla, CA, USA.; ^3^ Department of Neurosciences, University of California San Diego, La Jolla, CA, USA.; ^4^ Halıcıoğlu Data Science Institute, University of California San Diego, La Jolla, CA, USA.

## Abstract

During learning, synaptic connections between excitatory neurons in the brain display considerable dynamism, with new connections being added and old connections eliminated. Synapse elimination offers an opportunity to understand the features of synapses that the brain deems dispensable. However, with limited observations of synaptic activity and plasticity in vivo, the features of synapses subjected to elimination remain poorly understood. Here, we examined the functional basis of synapse elimination in the apical dendrites of L2/3 neurons in the primary motor cortex throughout motor learning. We found no evidence that synapse elimination is facilitated by a lack of activity or other local forms of plasticity. Instead, eliminated synapses display asynchronous activity with nearby synapses, suggesting that functional synaptic clustering is a critical component of synapse survival. In addition, eliminated synapses show delayed activity timing with respect to postsynaptic output. Thus, synaptic inputs that fail to be co-active with their neighboring synapses or are mistimed with neuronal output are targeted for elimination.

## Teaser

Spine elimination during motor learning is characterized by mistimed activity relative to both nearby synapses and spiking output.

## INTRODUCTION

Learning involves modifications of synaptic connections such that new connections are formed and some preexisting connections are lost. The change in the synaptic input repertoire of a given neuron results in modified response properties and thus participation in neural circuits.

Most excitatory synapses form on dendritic spines: small protrusions emanating from postsynaptic dendrites. Dendritic spines are highly dynamic, and their structure and strength can change dramatically in response to different patterns of synaptic activity. Numerous studies have identified patterns of synaptic input that either strengthen or weaken spine synapses, providing important clues about what types of synaptic activity are selected for by different modes of plasticity. For example, it has been suggested—using artificial suppression of neural activity—that inactive synapses are more likely to be eliminated, supporting a “use it or lose it” framework of synapse survival (*1*–*4*). However, evidence from both the developing central nervous system and neuromuscular junction suggests that inactive synapses are lost only when other inputs to the same postsynaptic target are active; i.e., global inactivity is insufficient for synapse elimination and can actually delay or prevent it (*1*–*3*). In addition, it has also been suggested that synaptic inputs correlated with postsynaptic activity are preserved, while those uncorrelated are eliminated, supporting an “out of sync, lose your link” scenario (*5*–*7*). The dominant model that has emerged from these findings is that synapses undergo heterosynaptic competition where active synapses capable of driving postsynaptic activity survive and trigger the elimination of relatively inactive synapses that are less capable of driving neural activity.

Several lines of evidence now support ample heterosynaptic interactions between plasticity events in a single neuron. For example, the induction of long-term potentiation (LTP) can lower the threshold for LTP at nearby spines and increase the likelihood of nearby spinogenesis, which may explain the observation that these events spatially cluster on individual dendrites (*8*–*12*). Further, it is well established that the induction of LTP can lead to heterosynaptic long-term depression (LTD) or elimination of inactive synapses on the same postsynaptic neuron (*13*–*15*). Competitive heterosynaptic interactions that balance the strengthening of active inputs with the weakening of inactive inputs are therefore commonplace in the nervous system and might represent a generalizable framework of destructive forms of plasticity, possibly including elimination. However, the paucity of direct observations of activity and stability of individual synapses in vivo—especially in the context of learning—presents challenges for extending these mechanisms to the intact adult brain. In particular, most studies have examined synapse elimination in the context of early development, or in analogous preparations (i.e., organotypic cultures), when plasticity mechanisms may be different from those in the adult brain (*16*). Furthermore, these studies often use artificial activation and/or silencing of inputs to examine the activity and plasticity dependence of synapse elimination. Thus, it remains unclear how physiological levels of activity and plasticity affect synapse elimination in the adult brain during learning.

Here, using longitudinal in vivo two-photon imaging of dendritic spine structure and function on apical dendrites in L2/3 excitatory neurons in the primary motor cortex (M1) during weeks of motor learning in mice, we investigated predictors of learning-related spine elimination. In contrast to previous reports in slice experiments, we find no evidence that eliminated spines are less active than stable ones, or that elimination is locally facilitated by other forms of plasticity. Instead, we find that the most distinctive functional feature of eliminated spines is their blunted co-activity with neighboring spines. Using simultaneous optical monitoring of synaptic glutamate and global calcium, we also show that eliminated spines’ activity is differentially timed with respect to postsynaptic activity. Our data support a model in which inclusion in locally coherent activity and contribution to postsynaptic activity is a central criterion for synapse survival. Together with the observations that locally coherent synaptic inputs can cooperate supra-linearly with the potential to strongly drive postsynaptic activity (*17*–*19*), we propose that synapse elimination contributes to the functional clustering of behaviorally relevant inputs, enhancing the robustness of learned circuits.

## RESULTS

**Fig. 1.**
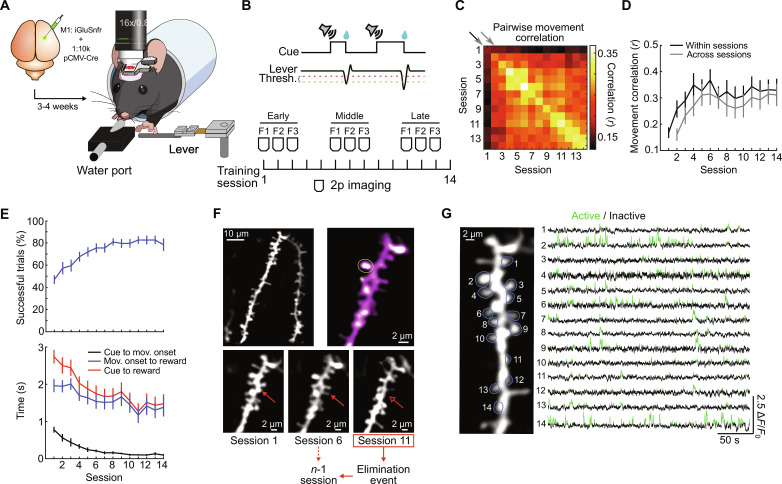
Motor learning task and in vivo functional synaptic imaging. (**A**) Schematic of experimental setup. (**B**) Top: Task structure. Bottom: Imaging and training schedule. (**C**) Correlogram of lever trajectory correlation within and across sessions (*n* = 24 mice). Each box corresponds to the median pairwise correlation coefficient of rewarded movement trajectories over a 3-s window. (**D**) The mean lever trajectory correlation increases both within [black line; center diagonal from (C); *P* = 0.016, Pearson’s correlation] and across [gray line; +1 diagonal from (C); *P* = 0.016, Pearson’s correlation] learning sessions (*n* = 24 mice). (**E**) Top: Percentage of trials resulting in reward increases over learning (*P* = 5.5 × 10^−15^, Pearson’s correlation). Bottom: Reaction time (black) as well as time from cue to reward (red) and movement onset to reward (blue) decrease over learning (*P* = 8.2 × 10^−23^ for reaction time; *P* = 1.5 × 10^−10^ for cue to reward; *P* = 5.5 × 10^−5^ for movement onset to reward; Pearson’s correlation). (**F**) Example apical dendrites of an L2/3 neuron in M1. Top left: Average projection of iGluSnFR2 fluorescence from an early imaging session. Top right: Overlaid average projection of fluorescence intensity from all frames (magenta) and from the frames in which the target spine (yellow circle) was active (green). Bottom: Zoomed-in image of the same dendrite over early, middle, and late sessions, highlighting the occurrence of spine elimination (red arrow). (**G**) Example iGluSnFR2 fluorescence traces of a subset of spines on the dendrite in (C). Portions of each trace classified as “active” are in green.

To investigate the structural and functional properties of synapses that predict their elimination during learning, we used a previously published dataset where we performed longitudinal two-photon imaging of synaptic function and structure in L2/3 excitatory neurons in M1 (*9*). To achieve this, we used the fluorescent glutamate reporter iGluSnFR2, which reports presynaptic glutamate release. We use it as a proxy for synaptic activity given its similar binding affinity to glutamate as AMPA receptors (*20*) and the fact that the release of a single vesicle is sufficient to activate postsynaptic AMPA receptors (*21*). However, we note that iGluSnFR2 does not directly reflect the magnitude of postsynaptic depolarization. Furthermore, it is possible that a subset of spines imaged are silent synapses, which may not be depolarized upon glutamate release. This is unlikely, however, as silent synapses are primarily located at filopodia (*22*), which we are not able to visualize in vivo (*9*). In addition, due to its relatively high baseline fluorescence, iGluSnFR2 can also be used to accurately visualize the structure of dendrites and dendritic spines, which we previously validated using correlated light and electron microscopy (CLEM) and other methods (*9*). This allowed us to longitudinally track the functional activity and structural stability of spines over the course of weeks as mice underwent a well-established motor learning task (Fig. 1 and fig. S1). The data for these experiments include those previously published (21 mice) (*9*) and those from new experiments (3 mice). Briefly, in this task, water-deprived mice learn to press a lever in response to an auditory cue to receive a water reward (Fig. 1B). With daily training, mice showed a significant improvement in movement correlation, success rate, and reaction time over 2 weeks (Fig. 1, C to E). Throughout training, we imaged up to three fields of view (FOVs) per animal containing the apical dendrites of L2/3 excitatory neurons in M1 on different imaging sessions. To minimize photodamage, we imaged each FOV three times, with a 5-day interval between imaging days for a given FOV, which we have previously shown to be well tolerated by dendrites (*9*). We specifically targeted L2/3 excitatory neurons in M1, as these cells have been shown to display robust plasticity that coincides with the development of the learned movement pattern (LMP) in this task (*23*–*25*). Specifically, the apical dendrites of these neurons display a burst of spine formation and spine elimination, coinciding with an expansion and subsequent contraction of the active movement-related population of neurons (*23*). Manipulations that block spine turnover also impede the development of a reproducible motor pattern, supporting the idea that such plasticity is necessary for the repetition-mediated refinement of motor behaviors (*26*). By tracking synaptic activity and structure using the genetically encoded fluorescent glutamate indicator iGluSnFR2, our recent work also revealed that new spines formed during this task encode learned behaviors, largely through co-activation with nearby spines (*9*). The collective of these findings supports motor learning as a reliable platform to investigate the synaptic substrates of learned behaviors.

### Eliminated spines spatially cluster with movement-related inputs, but not other forms of plasticity

We sought to define predictors of spine elimination on the apical dendrites of L2/3 excitatory neurons in M1 during motor learning. By visualizing apical dendrites over multiple days of learning, we identified spines that were eliminated (8.08% of all spines imaged) across imaging sessions (Materials and Methods; Fig. 1F). This approach allowed us to characterize structural and functional properties of spines that give rise to spine elimination.

**Fig. 2.**
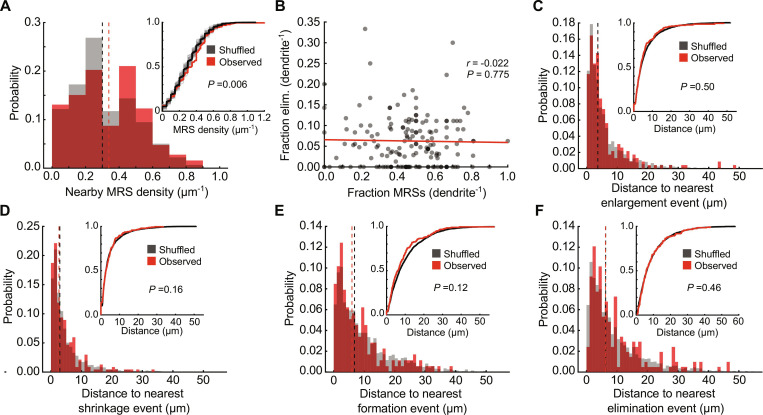
Dendritic spines that are eliminated over motor learning do not spatially cluster with other plasticity events. (**A**) Spine elimination occurs in areas of higher MRS density. Histogram of the density of MRSs within 10 μm of an elimination event (red) compared to shuffled values (gray). Medians are shown in vertical lines. Inset: Cumulative probability plot of data shown in histograms. *P* value represents the fraction of shuffles that did not conform to the hypothesis that MRS density nearby eliminated spines is greater than chance. *n* = 24 mice/252 eliminated spines/1929 MRSs. (**B**) The fraction of eliminated spines does not correlate with the fraction of MRSs on individual dendrites. Statistics represent Pearson’s correlation coefficient. *N* = 24 mice/162 dendrites. (**C**) Spine elimination is not spatially clustered with enlargement events. Histograms show the distances between the elimination event and the nearest enlargement event (red) compared to shuffled data (gray). Inset: Cumulative probability plot of data shown in histograms. *P* value calculated as in (A). *n* = 24 mice/252 eliminated spines/693 enlarged spines. (**D**) Same as (C), but for shrinkage events. *N* = 24 mice/252 eliminated spines/923 shrunken spines. (**E**) Same as (C), but for spine formation events. *N* = 24 mice/252 eliminated spines/179 new spines. (**F**) Same as (C), but for other elimination events. *N* = 24/252 eliminated spines.

We previously reported that spine formation in this task is enriched in the local dendritic environment with the potentiation of preexisting spines that were significantly more active during movement periods than other periods [i.e., “movement-related spines” (MRSs); Materials and Methods] (*9*). Thus, we first asked whether there is also an enrichment of movement-related information near sites of elimination on the sessions before a given elimination event. Similar to new spines, we found that the density of MRSs nearby elimination events was higher than expected by chance (estimated by shuffling the labels of eliminated and stable spines; Materials and Methods) (Fig. 2A), suggesting that local convergence of movement-related information locally enhances the probability of spine elimination. It is possible that this result arises due to dendrite-wide enrichment of MRSs and eliminated spines. However, we found no correlation between the fraction of MRSs and fraction of eliminated spines of individual dendrites (Fig. 2B) arguing against this possibility. Thus, it appears that elimination is more likely to occur in local regions along individual dendrites enriched in movement-related information.

Next, we asked whether spine elimination spatially clusters with other forms of plasticity. We considered several spine plasticity events, including the enlargement (1.5×) or shrinkage (0.75×) of preexisting spines [suggesting potentiation and depression, respectively (*27*–*28*)], as well as the formation of new spines and other spine elimination events. We performed nearest-neighbor analysis, asking whether the nearest spine plasticity event (i.e., enlargement, shrinkage, formation, or elimination, considered independently) to each eliminated spine was closer than expected by chance. We did not find any evidence that spine elimination spatially clusters with, or avoids, these forms of spine structural plasticity (Fig. 2, C to F). These results were consistent across a range of different thresholds for spine enlargement and shrinkage (fig. S2, A and B), indicating that this result is not sensitive to the selected plasticity thresholds. This result also held when considering the plasticity of all spines or of MRSs only (fig. S2, C and D). Last, the spines eliminated between early and middle sessions (early elimination events) and the spines eliminated between middle and late sessions (late elimination events) showed consistent results (fig. S2, E to I). Thus, unlike the addition of new spines, the elimination of preexisting spines throughout the course of motor learning does not appear to locally interact with the plasticity state of other nearby spines on the same dendrite. However, we acknowledge that our results do not exclude all potential interactions between elimination and plasticity events, such as those with longer length scales than what we examined.

### Spine elimination is unlikely to occur due to disuse

**Fig. 3.**
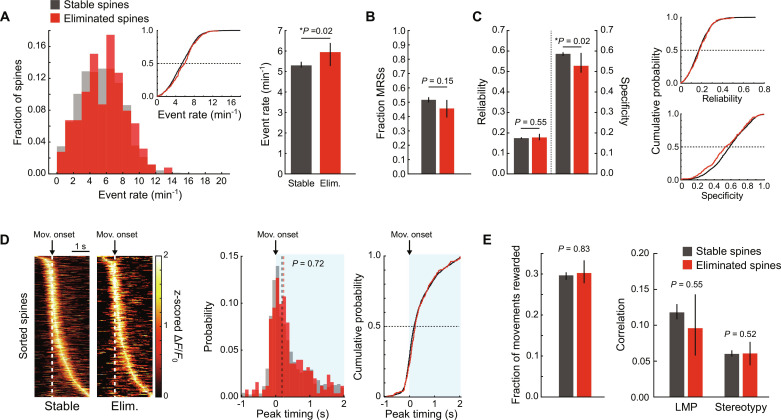
Spine elimination during motor learning is unlikely to occur due to disuse or lack of task engagement. (**A**) Left: Histograms of event rates between eliminated (red) and stable (gray) spines. Inset: Corresponding cumulative probability plot. Right: Bar summary. Median ± bootstrapped 95% confidence intervals for this and all other bar graphs. Eliminated spines display significantly higher event rates (*P* = 0.02, mixed-effects model). (**B**) Eliminated spines show comparable probability of being MRSs. Summary of the fraction of MRSs for stable (gray) and eliminated (red) spines (*P* = 0.15, mixed-effects model). (**C**) Eliminated spines have similar movement reliability (*P* = 0.55, mixed-effects model) but have a lower movement specificity (*P* = 0.02, mixed-effects model). Left: Summaries of reliability and specificity for stable and eliminated spines. Right: Cumulative probability plots for the data. (**D**) Eliminated spines and stable spines show comparable population activity profiles during lever movements. Left: Heat map of *z*-scored mean Δ*F*/*F*_0_ values during lever presses for all spines aligned to movement onset, sorted by peak time. Middle: Histograms of peak activity timing for stable (gray) and eliminated (red) spines. Median values are shown as vertical dashed lines (*P* = 0.72, mixed-effects model). Right: Cumulative probability plot. (**E**) Eliminated spines and stable spines encode movements of comparable outcome and kinematic quality. Left: Fraction of movements that were rewarded coincident with a given spine’s activity. Middle: Correlation of all movements coincident with the activity of a given spine with the learned movement pattern (LMP) (*P* = 0.55, mixed-effects model). Right: Stereotypy of encoded movements. *n* = 24 mice/2671 stable spines/252 eliminated spines.

Given that spine elimination does not spatially cluster with other plasticity events, we next explored functional features of individual spines that might predict their own elimination. It has previously been shown that during development less active synapses tend to be eliminated, while more active synapses are stabilized (*3*). Thus, we first considered the possibility that spines eliminated during motor learning were less active than stable spines. To address this possibility, we characterized the frequency of activity “events” [i.e., periods when the iGluSnFR2 signal rose above a noise-based threshold, as previously described (*9*)] in both stable spines and spines that were eliminated by the subsequent imaging session. We found no evidence that eliminated spines are less active than stable spines (Fig. 3A and fig. S3A). Instead, eliminated spines display activity event rates that are slightly but significantly higher than that of stable spines, eliminating the possibility that inactivity is a primary driver of spine elimination in this context. Given this result, we next asked whether eliminated spines, while active, were less engaged in the task. To this end, we first compared the fractions of stable and eliminated spines that met the criterion for categorization as MRSs. Despite a trend toward eliminated spines being less likely to be categorized as MRSs, this difference did not reach significance (Fig. 3B and fig. S3B). Similarly, we found no evidence that eliminated spines were differentially related to any other task feature that we explored (fig. S3C).

To understand more about the relationship between eliminated spines’ activity and movements, we measured how reliably spines were active during movement periods (i.e., the fraction of movements coincident with a given spine’s activity) as well as how specific their activity is to movement periods (i.e., the fraction of a given spine’s activity that is coincident with movements). We found that while the movement reliability of spine activity is comparable between stable and eliminated spines, the movement specificity of spine activity is significantly lower for eliminated spines (Fig. 3C and fig. S3D). Thus, while eliminated spines are reliably activated during lever-movement periods, they are more likely to display “off-target” activity occurring outside of lever-movement periods. These results, coupled with the higher activity rates displayed by eliminated spines, raise the possibility that eliminated spine activity occurs more indiscriminately with respect to movements. To address this, we aligned spine activity to movement onset and compared the temporal distributions of peak activity between the stable and eliminated populations (Fig. 3D). Because spine activity is sparse and activity occurs during only a subset of movements, we only considered movements during which a given target spine was active. Despite the significantly lower movement specificity, we found that eliminated spines show temporal distributions of peak activity surrounding movement onset that were very similar to stable spines such that most spines were biased toward peaking soon after movement onset, similar to the reported neuronal activity of excitatory neuron somata in L2/3. Median peak activity times were indistinguishable between the two populations (Fig. 3D). Thus, eliminated spines’ activity is not random with respect to movement timing. In contrast to previous findings from development, these results imply that eliminated spines are active and engaged in motor encoding during the task, although they are more likely to be also active during nonmovement periods.

Given the overall similarity of movement-related activity of stable and eliminated spines, we next considered the possibility that the patterns of movements encoded by eliminated spines are different than those encoded by stable spines. To address this, we considered three features of executed lever movements related to the learning of this task. First, we measured the fraction of rewarded movements out of all the movements during which each spine was active. Both stable and eliminated spines showed rewarded movement fractions of ~30%, and were not significantly different, indicating that eliminated spines are just as likely to be active during rewarded movements (Fig. 3E). Next, given that a central feature of motor learning is the development of more reproducible movement kinematics over time, we inspected two metrics designed to account for the kinematic similarity of lever movements encoded by spines. First, we considered how much each movement resembled the LMP, defined as the average lever trace displayed by each animal in the late sessions (*11*–*14*) of learning (*9*, *23*). We reasoned that eliminated spines might encode movements that, while equally likely to result in a reward, were kinematically distinct from the learned pattern and would therefore show lower correlation values with the LMP. However, the LMP correlations of the movements during which eliminated spine were active were not significantly different from the stable population (Fig. 3E). We then considered the possibility that stable spines might be active during movements that are more self-similar—or “stereotyped”—than those encoded by eliminated spines. However, the correlation among movements was not different between the stable spine activity and eliminated spine activity (Fig. 3E). Together, these results suggest that eliminated spines encode movements of comparable quality to stable spines.

### Eliminated spines are structurally smaller synapses

Several studies have found that the probability of spine elimination is related to spine structure: Larger spines are more likely to survive, and smaller spines are more likely to be eliminated (*29*–*31*). Consistent with these reports, we found that eliminated spines are significantly smaller than stable spines (fig. S4A). Given the strong correlation between spine size and synaptic strength, this implies that most spines selected for elimination during learning are already weak synapses. We therefore sought to understand whether any observed distinguishing features of eliminated spines’ function could be explained by their size alone. In support of this possibility, we observed a significant negative correlation between the spine area and iGluSnFR2 event rates among stable spines such that smaller spines display higher event rates (fig. S4B). Because both eliminated spines and smaller stable spines display higher event rates, we performed additional analyses comparing eliminated spines to small stable spines. We defined “small” stable spines as those below the 40th percentile of stable spine areas, yielding a subpopulation of stable spines whose areas are not significantly different from those of the eliminated population (Materials and Methods, fig. S4C). We found that eliminated spines are not significantly more active than small stable spines, suggesting that the observed difference in event rates can be explained more by spine size than by spine stability (fig. S4D). Similarly, the movement specificity of eliminated spines’ activity is not different than that of small stable spines (fig. S4, E and F). These results reveal that eliminated spines are typically smaller than stable spines, and that this smaller size is likely sufficient to explain some of the apparent functional differences at the level of individual spines. In addition, we observed that both early eliminated and late eliminated spines are smaller than stable spines (fig. S4, G to I), indicating that throughout the course of learning smaller spines are preferentially eliminated.

### Eliminated spines display weak functional clustering

**Fig. 4.**
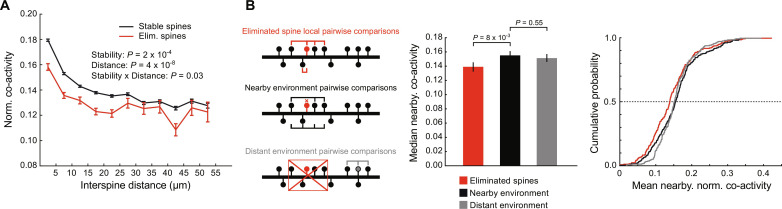
Eliminated spines display lower functional clustering. (**A**) Eliminated spines show lower co-activity with neighboring spines. Curves show normalized co-activity rates of all spine pairs as a function of the dendritic distance between them. Interspine distances are grouped in 5-μm bins. All spines display stronger co-activity with nearby spines (mixed-effects model, fixed effect of distance: *P* = 1 × 10^−7^). Eliminated spines show lower overall co-activity rates with other spines (fixed effect of spine stability: *P* = 0.02), especially with nearby spines (fixed effect of stability × distance interaction: *P* = 0.03). (**B**) The lower co-activity displayed by eliminated spines is not due to being in dendritic regions of lower co-activity. Left: Schematic of three groups used to assess the co-activity levels of eliminated spines versus their local and more distal environments. The pairwise co-activity between an eliminated spine (red) and all of its neighbors within 10 μm defines the eliminated spine score (top). The nearby environment is defined as the pairwise co-activity between all spines neighboring an eliminated spine, but excluding the eliminated spine itself (middle). The distant environment is defined as the co-activity levels of spines >10 μm from an eliminated spine with their neighboring (≤10 μm) spines. Middle: Bar graph summaries of mean nearby co-activity levels for the different categories of spines. The nearby environment and distant environment groups were averaged for each eliminated. Median ± 95% confidence intervals. Eliminated spines (red) are less co-active with their nearby spines than the other spines in the nearby environment are with each other (black) (*P* = 0.8 × 10^−3^, mixed-effects model). The nearby environment group shows comparable co-activity levels to the distant environment group (*P* = 0.55, mixed-effects model). Right: Cumulative probability plots of the groups shown at left. *n* = 252 eliminated spines.

In our previous work, we showed that spines that form during learning display correlated activity with nearby task-related spines, contributing to functional clustering of task-related inputs (*9*). We hypothesized that the elimination of preexisting spines might also subserve functional clustering by removing desynchronized inputs. To investigate this possibility, we compared normalized co-activity rates of stable and eliminated spine pairs as a function of interspine distance, as previously described (*9*). Consistent with our hypothesis, we found that eliminated spines are significantly less co-active with other spines on the same dendrites, especially at closer distances (Fig. 4A). This observation was also true when considering correlation coefficients of their activity, illustrating that this outcome is robust to the metric used to evaluate the activity similarity of spine pairs (fig. S5). These results were unlikely due to the smaller sizes of eliminated spines, as small stable spines displayed functional clustering curves that were indistinguishable from large stable spines (fig. S6A). In addition, both early and late eliminated spines displayed lower co-activity levels compared to stable spines (fig. S6B) and the co-activity levels of late eliminated spines were lower throughout training before their elimination (fig. S6C).

The lower co-activity of eliminated spines could arise from eliminated spines existing in dendritic domains that show lower overall co-activity. Alternatively, eliminated spines may be uniquely uncorrelated with their neighboring spines. To distinguish these possibilities, we assessed the mean pairwise co-activity of each eliminated spine with all its neighboring (≤10 μm) spines and compared this value to the mean co-activity displayed within these same groups of neighboring spines when excluding the eliminated spine (Fig. 4B). We found that eliminated spines are indeed less co-active with their neighboring spines than their neighboring spines are with each other, suggesting that the blunted functional clustering observed in eliminated spines is not merely a by-product of them existing in dendritic regions with low co-activity (Fig. 4B). Further, we observed no significant difference between the mean co-activity of spines neighboring an elimination event and the local co-activity of spines farther (>10 μm) from elimination events (Fig. 4B). We thus conclude that eliminated spines are uniquely desynchronized with their dendritic environments, which otherwise display comparable co-activity levels to other dendritic locations.

Together, these results show that eliminated spines display markedly reduced functional clustering compared to stable spines. Such results draw clear parallels with “transient” new spines, which display blunted functional clustering and are eliminated shortly after formation (*9*). Thus, maintaining coherent activity with nearby spines might be a generalizable criterion for spine survival during motor learning.

### Eliminated spines show mistimed activity relative to postsynaptic output

**Fig. 5.**
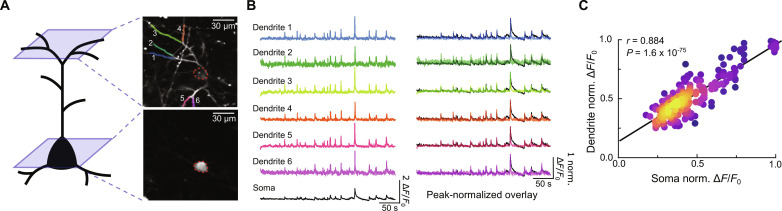
Global dendritic calcium events reflect somatic activity. (**A**) Schematic and representative image of near-simultaneous dual-plane recordings of dendrites and somata from RCaMP2-expressing L2/3 neurons. Colored/numbered dendrites correspond to traces shown in (B). (**B**) Left: Example Δ*F*/*F*_0_ traces of RCaMP2 of dendrites (top, color-coded) and their shared parent soma (bottom, black). Right: Same Δ*F*/*F*_0_ traces normalized to their own peak overlaid with somatic traces. (**C**) Dendritic and somatic Ca^2+^ events are tightly coupled in L2/3 neurons in M1. The amplitude of normalized Ca^2+^ events in dendrites plotted against the amplitude at the soma. Each point represents a single dendritic event. Points are color-coded using a kernel density function. Statistics correspond to Pearson’s correlation coefficient. *n* = 2 mice/4 neurons/15 soma-dendrite pairs/225 total paired events.

Functionally clustered inputs likely have a disproportionately strong impact on postsynaptic function due to supra-linear summation of nearby and simultaneous synaptic inputs. A converse of this idea is that eliminated spines that show weak functional clustering might be less capable of driving postsynaptic activity. To test this hypothesis, we performed additional experiments to examine the relationship between synaptic inputs and cellular activity of the postsynaptic neuron. We did this by sparsely coexpressing the improved glutamate sensor iGluSnFR3 (*32*) (fig. S1), along with a red-shifted calcium indicator, RCaMP2 (*33*), to simultaneously visualize synaptic activity alongside postsynaptic calcium (Materials and Methods). On the basis of a previous report (*34*), we reasoned that large dendritic calcium events in L2/3 neurons probably reflect the spiking activity of postsynaptic neurons. We validated this approach by performing near-simultaneous two-plane imaging of dendrites and somata of L2/3 excitatory neurons coexpressing iGluSnFR3 and RCaMP2 to assess the coincidence of dendritic and somatic calcium events (Materials and Methods; Fig. 5A). Comparisons between the RCaMP2 fluorescence of dendrites and their parent somata revealed a virtually complete coincidence between calcium events across these compartments (Fig. 5B). Furthermore, the amplitudes of dendritic and somatic events were strongly and significantly correlated, with no obvious indication that dendritic events occurred in the absence of somatic events (Fig. 5C and fig. S7, A and B). Further, sister dendrites from the same neuron were also highly correlated with each other, suggesting that branch-specific events—if they occur—are rare (fig. S7, C to E). These findings are consistent with a previous report showing that most dendritic calcium events correspond to back-propagating action potentials (APs) in L2/3 neurons of the secondary motor cortex (*33*). Together, these results support the use of L2/3 dendritic calcium events as a proxy of neural output.

**Fig. 6.**
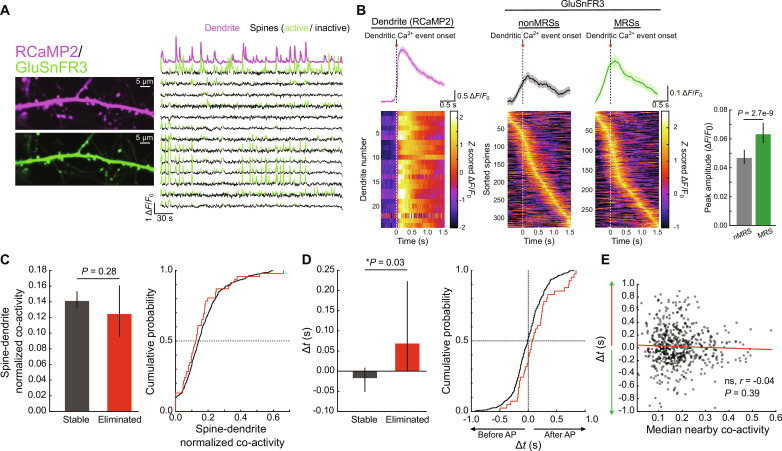
Simultaneous monitoring of spine glutamate and dendritic calcium shows that eliminated spines shows delayed activity timing relative to postsynaptic activity. (**A**) Example in vivo two-photon images of iGluSnFR3 (green) and RCaMP2 (magenta) coexpressing neurons. Left: Average projection images of the two sensors from the same dendrite. Right: Example Δ*F*/*F*_0_ traces of dendritic RCaMP2 (magenta, top) alongside several of its spines. For spines, events are shown in green. (**B**) The relationships of spine activity with dendritic calcium events reflect task-relatedness of individual spines. Left: Average dendritic calcium events. Top: Average RCaMP2 Δ*F*/*F*_0_ trace aligned to dendritic event onset. Bottom: Heat map of individual dendrites sorted by peak time. Middle: Average iGluSnFR3 traces aligned to dendritic Ca^2+^ event onset, separated into non-MRSs (left) and MRSs (right). Right: Peak amplitudes of MRS iGluSnFR3 (green) events are greater than those of non-MRSs (gray) when dendrites are active. Median ± bootstrapped 95% confidence intervals. *P* = 2.7 × 10^−9^, rank-sum test. (**C**) The normalized co-activity rate between spines and dendrites is similar for stable and eliminated spines. Left: Bar summary of the normalized co-activity between spines and dendrites for stable (gray) and eliminated (red) spines. Median ± bootstrapped 95% confidence intervals. Right: Cumulative probability plot of the data. (**D**) Eliminated spines show delayed activity during dendritic calcium events compared to stable spines. Left: Bar summaries of Δ*t* values for stable (gray) and eliminated (red) spines. Median ± bootstrapped 95% confidence intervals. Right: Cumulative probability plot of the data. Dashed lines represent the median (horizontal) and Δ*t* = 0 (vertical). *P* = 0.03, mixed-effects model. (**E**) Spine-dendrite relative AP timing (Δ*t*) does not correlate with the spine’s nearby co-activity levels. Statistics represent Pearson’s correlation coefficient.

We next assessed the relationship between the activity of individual spines with neural outputs as mice underwent motor learning (Fig. 6A and fig. S8). As a general description of this relationship, we aligned spine activity to onset of dendritic calcium events. This showed a clear relationship between synaptic inputs and spiking output, with the average synaptic inputs rising before calcium activity (Fig. 5B). MRSs show an activity pattern that is more strongly locked to calcium activity, indicated by greater event amplitudes (Fig. 6B), consistent with the notion that a primary drive of M1 L2/3 neurons is related to ongoing movements. These results give us confidence that this coexpression system reports expected relationships between task-related synaptic input and postsynaptic output.

Before comparing stable and eliminated spines, we first validated that iGluSnFR3 also provides a reliable readout of dendritic structure. Similar to our observation with the previous generation of iGluSnFR2 (*9*), estimation of spine area and its changes across days is not affected by spine activity (fig. S9, A and B). In addition, we compared structural images captured using 925-nm excitation wavelength typically used for functional imaging with those captured using 810-nm wavelength, which is near the isosbestic point of iGluSnFR3 where its fluorescence is independent of glutamate concentration (*32*) and therefore reflects a pure structural signal (fig. S9C). We found that all spines observed at 810 nm were still visible at 925 nm, and the spine areas estimated from these two wavelengths strongly correlated (fig. S9, C and D), supporting the use of iGluSnFR3 as a reporter of dendritic structure.

To test whether eliminated spines show a differential relationship with neural output, we compared the normalized co-activity rates between spine iGluSnFR3 events and dendritic RCaMP2 events (analogous to our spine-pair co-activity measurements). We found that eliminated spines show slightly lower spine-dendrite co-activity levels than stable spines, but the difference did not reach statistical significance (Fig. 6C). These results held when considering either correlation coefficients of spine-dendrite activity or the fraction of spine events coincident with dendritic events, and were not influenced when considering when spines were eliminated (early versus late) (fig. S10). Thus, despite the lower functional clustering observed in eliminated spines, this difference does not extend to a systematic difference in the likelihood that their activity occurs during neural output. We note, however, that these co-activity metrics are largely agnostic to the relative timing of spine activity and neural output, which has been consistently proposed as a critical component of Hebbian plasticity mechanisms (*35*–*36*). Such a Hebbian framework would predict that synapses undergoing depression-like phenotypes (possibly including spine elimination) would be more consistently active after dendritic event onset. While the relatively poor temporal resolution of fluorescent indicators precludes an exact measurement of absolute timing of events, we reasoned that consistent relative differences between spine populations may be detectable. We therefore compared the relative timing of iGluSnFR3 event onsets with respect to dendritic event onsets in both stable and eliminated spines. We found that the relative onset of eliminated spines’ activity was significantly delayed compared to stable spines (Fig. 6D). This difference appeared to primarily occur during the later phases of training (fig. S11, A and B), although this did not reach statistical significance, possibly due to small sample sizes. This effect was again unlikely to be due to the small sizes of eliminated spines, as we observed no significant correlation between spine area and relative timing among stable spines (fig. S11C), and small stable spines still showed significantly earlier onset than eliminated spines (fig. S11D). These results suggest that spines whose inputs are more delayed relative to the postsynaptic neuron’s spiking are more likely to be eliminated.

We next asked whether the two primary functional predictors of spine elimination—namely, their weaker functional clustering and their lagged timing with respect to dendritic events—are related parameters. We hypothesized that spines showing strong functional clustering would be more likely to drive neural output, with their activity more consistently preceding neural output. However, we observed no significant correlation between spines’ relative timing with dendritic events and their local co-activity levels (Fig. 6E). Thus, our data suggest that functional clustering and relative event timing are independent contributors to a spine’s probability of elimination.

## DISCUSSION

The removal of certain streams of information is a critical component of refining neural circuits in the nervous system. During development, such elimination permits the establishment of specific connectivity, allowing for the proper wiring of neural circuits to subserve specific functions, such as the formation of motor units in the periphery and ocular dominance columns in the cortex. In the adult brain, synapse elimination remains prominent, with numerous lines of study indicating an important role in natural learning and memory (*37*) while also being linked with various neurological disorders (*38*). Thus, synapse elimination appears to be critical not only for the establishment of neural circuits but also for their continual modification throughout adulthood.

While many studies have examined the mechanisms underlying synaptic elimination during early development, the processes governing experience-dependent synapse elimination in the adult brain, especially those responsible for selecting which synapses are pruned away, remain poorly understood. Here, we used longitudinal in vivo two-photon imaging of spines during learning to understand how their structural and functional features designate them for elimination. We found no evidence that spine elimination spatially clusters with other forms of plasticity on the same dendrite, suggesting that the mechanisms underlying spine elimination in this context do not strongly interact with mechanisms controlling other forms of plasticity, or even with other elimination events. This result was surprising, given the numerous forms of heterosynaptic plasticity previously reported (*1*, *8*, *10*, *13*, *14*, *39*–*42*), with one such study suggesting that LTD and spine elimination may be inherently heterosynaptic phenomenon, provided sufficient gamma-aminobutyric acid (GABA)-ergic inhibitory input (*42*). One possible explanation for the discrepancy between our current findings and previous reports is the notable disinhibition (removal of inhibitory inputs from somatostatin-expressing interneurons) observed at the apical dendrites of L2/3 neurons in M1 during motor learning (*26*), which might minimize the influence of GABA-driven forms of plasticity. Thus, the heterosynaptic properties of spine elimination may be heavily context dependent, differing across brain areas, cell types, subcellular domains, and/or the particular milieu of input types. We note, however, that it is possible that heterosynaptic effects with longer length constants were missed in our study due to the limited lengths of dendrites accessed in each image field.

Functionally, we found no evidence that eliminated spines were inactive or disengaged with respect to the motor task, suggesting that experience-dependent spine elimination does not occur due to the disuse of the synapse. This is in stark contrast to findings from early development, where relatively inactive synapses are more likely to be eliminated (*1*–*4*), and argues against a simple use it or lose it model for experience-dependent synapse elimination in adult cortex. Instead, we found that eliminated spines display weaker local co-activity with other nearby spines. This suggests that it is the coherence of a synapse’s activity with others, rather than its individualized activity, that regulates its stability, and, to our knowledge, represents the first demonstration of such a mechanism regulating synapse elimination. It is worth noting, however, that a previous study has shown that low coactivity rates may promote presynaptic depression of synapses, a distinct plasticity event from elimination, during early development (*43*). In addition, this observation mirrors our previous findings that newly formed spines in this paradigm are more likely to survive when they are co-activity with nearby spines (*9*). Together, this work suggests that dendrites may inherently favor locally synchronized synaptic inputs. Such functional clustering has emerged as an attractive framework for the organization of synapses, owing in part to the possibility that locally co-active synapses can induce voltage-dependent supra-linearities in dendrites, thereby granting clustered synapses disproportionately strong weight in their contribution to neuronal output. Therefore, by removing out-of-sync inputs, spine elimination may support the functional clustering of synapses along dendrites important for neural computations.

Beyond local dendritic mechanisms, a large body of work has indicated that the global activity of a neuron is important in shaping the plasticity of its synapses (*35*, *36*). By simultaneously monitoring synaptic activity and neural output, we found that eliminated spines overall are similarly active during neural output compared to other spines. However, we found that the onset of activity for eliminated spines with respect to neural output was relatively more delayed compared to stable spines. We emphasize that, due to the poor kinetics of the fluorescence sensors used here, we cannot determine the absolute timing of these events relative to neural output. Nevertheless, these results are reminiscent of spike timing-dependent plasticity (STDP) mechanisms (*35*, *36*). We found that the activity timing with respect to neural output did not correlate with local co-activity levels, suggesting that activity timing with respect to neural output and nearby spines work independently to regulate spine elimination. Therefore, different mechanisms may be engaged at different times and in different contexts to trigger the elimination of synapses. Both of these mechanisms, however, likely serve the same end goal of selecting spines for survival with greater control over neural output, either through their participation in functional clusters or through the timing of their activity.

We also found that eliminated spines are smaller. This aligns well with slice studies suggesting that spines with smaller initial sizes are most likely to be eliminated during LTD induction (*28*) and in vivo studies showing the shorter lifetimes of small spines (*29*, *31*). As spine size strongly correlates with synaptic strength, this likely means that weaker synapses are at risk of elimination when learning commences. This is consistent with the notion that synapse elimination helps to select synapses with greater control over AP firing, as weaker synapses, especially those that are not functionally clustered, are less capable of depolarizing the soma.

To understand the functional relevance of experience-dependent synapse elimination and the mechanisms regulating it, it is important to consider it in the context of the behavioral and circuit adaptations that occur during learning. Over the course of training in our task, the movements of mice become more consistent and reproducible across trials and sessions, indicating the formation of a stable movement pattern. Most of the behavioral improvements appear to have occurred during the first week of training, and may suggest that synapse elimination during early training is more behaviorally relevant than later during training. However, we have previously shown that the representation of movements in L2/3 pyramidal neurons in M1 remains dynamic over the course of training (*23*), and synaptic plasticity, including synapse elimination, likely contributes to the continued reorganization of M1 circuitry. It is conceivable that synapse elimination may contribute to multiple facets of M1 reorganization. For example, synapse elimination may remove desynchronized inputs to reduce noise to a given neuron and produce more temporally consistent activity, and seen over the course of learning (*23*). Future studies manipulating synapse elimination, however, will be needed to determine the precise role of synapse elimination in the experience-dependent reorganization of neural circuits. In addition, while the mechanisms reported here are likely driven by learning due to the higher rates of spine elimination (*26*) and synaptic coactivity (*9*) seen during learning, similar mechanisms may be engaged in other contexts. This, however, will need to be determined in future studies.

One unanswered question is what happens to the presynaptic terminal of eliminated spines. One possibility is that the presynaptic terminal is also eliminated, resulting in the complete removal of the synapse. An alternative possibility is suggested by the previous observation by us and others that, during synaptogenesis, nascent synapses preferentially form contacts with multi-synaptic boutons that already form connections with preexisting spines (*9*, *44*–*46*). Considering that most mature synapses involve presynaptic boutons that are dedicated to the synapse, it seems likely that the connection of the multi-synaptic boutons with the preexisting spine is lost as the new synapse matures. Therefore, it is possible that the presynaptic terminals of eliminated spines persist, but form new connections with spines on other dendrites, which likely has differential effects on how information is transmitted through the circuit. Future studies, however, will be needed to determine the fate of the presynaptic terminals of eliminated spines.

Overall, the findings of this study support a model whereby both local and global mechanisms are engaged to determine the survival of individual synapses that contribute to controlling the output of the neuron. These mechanisms likely play a critical role in the remodeling of neural circuits to allow for the flexible adaptation of behavior throughout adulthood.

## MATERIALS AND METHODS

### Animals

All animal procedures were performed in accordance with guidelines set forth and protocols approved by the University of California San Diego Institutional Animal Care and Use Committee and the National Institutes of Health. Mice (C57BL/6) were group-housed in disposable cages with standard bedding in a temperature-controlled room with a reversed light cycle. All experiments were performed during the dark cycle. Males and females were randomly used for surgeries, with no selection criteria other than surgery outcome.

### Surgery

Surgical procedures were performed as previously described (*9*). Briefly, adult mice (6 weeks or older) were anesthetized with isoflurane in an enclosed, ventilated chamber (5% isoflurane with a constant flow rate of 1 liter/min at 0.1 bar and 21°C) until a deep plane of anesthesia was reached, as indicated by low muscle tone, slowed respiration rate, and lack of response to tail and toe pinch. Baytril (10 mg/kg) and dexamethasone (2 mg/kg) were injected subcutaneously to prevent infection and brain swelling, respectively. A craniotomy (~3 mm diameter) was performed, as previously described (*23*), over the right caudal forelimb area around the central coordinate of ~300 μm anterior and ~1500 μm lateral from bregma. Viruses (AAV1-CMV-PI-CRE, Addgene; AAV9-pCaMKII-Cre, Addgene; AAV1-Syn-FLEX-SF-iGluSnFR-A184S, construct received from L. Looger; AAV8-Syn-FLEX-GluSnFR3-GPI, VectorBuilder; AAV2/1-EF1a-FLEX-RCaMP2, Neurophotonics) were mixed so as to achieve sparse expression of iGluSnFR (1:1 mixture of undiluted iGluSnFR and 1:5000 to 1:20,000 pCMV-Cre diluted in saline + 0.5% FastGreen for visualization of injections) or GluSnFR3 + RCaMP2 expression (1.8:2:1 iGluSnFR3:RCaMP2: 1:10,000 diluted pCaMKII-Cre) and injected into the region of the caudal forelimb area of the exposed cortex using beveled glass pipettes (~12 to 25 μm inner diameter). Each injection consisted of a ~20-nl volume at a depth of ~250 μm from the pial surface to target layer 2/3. Injection volumes were dispensed over the course of ~2 min. Multiple (three to five) injections were performed in each craniotomy, separated by at least 500 μm. Pipettes were left in the brain for 4 min after injection to avoid backflow of virus. Chronic imaging windows consisting of a 3-mm-diameter plug glued to a larger (~5 mm) glass base were then implanted in the craniotomy. The window was held in place with gentle pressure, while the edges were affixed to the skull with small amounts of surgical glue (VetBond). Buprenorphine was injected subcutaneously at the end of surgery for pain management.

### Water restriction

Animals were allowed to recover from surgery for ~10 to 14 days, after which they were progressively water-restricted (2 ml/day for 3 days, 1.5 ml for 3 days, then 1 ml/day for the remainder) for ~14 days. Weight was monitored daily to ensure loss of no more than 30% starting body weight. Mice losing more than this amount of weight were administered double the prescribed daily amount of water until recovery.

### Behavior

Behavioral training was performed as previously described (*9*). Briefly, after water restriction, mice were trained in the lever press task for 14 days. Simultaneous two-photon imaging was performed on sessions 1 to 3 (“early sessions”), 6 to 8 (“middle sessions”), and 11 to 13 (“late sessions”). During each trial, a 6-kHz tone (“cue”) was presented to indicate a period (maximum of 10 s) during which a lever press could be rewarded with water. A successful lever press during this period triggered a 500-ms,12-kHz tone and the delivery of a water reward (~8 to 10 μl per trial), followed by an intertrial interval of 8 to 12 s. Successful lever presses were defined as those crossing two thresholds (~1.5 mm and ~3 mm below the resting position) within 200 ms. The 3-mm threshold defined the target lever displacement, while the 1.5-mm threshold ensured that the mouse did not hold the lever near the target threshold. Failure to perform a successful press during cue presentation triggered a white noise punishment signal and the start of the next intertrial interval. “Non-cued” presses during the intertrial interval were neither rewarded nor punished. Mice were exposed to ~100 trials each day, or until the mouse became disengaged (no movements for 20+ trials) or satiated (no licking in response to water delivery).

### In vivo two-photon imaging

Imaging was performed as previously described (*9*). Briefly, imaging was performed using a commercial two-photon microscope (B-Scope, ThorLabs) equipped with a 16×/0.8-NA (numerical aperture) objective (Nikon) and a Ti-Sa laser (Newport) tuned to 925 nm (for iGluSnFR2 imaging), 810 nm (for isosbestic imaging), or 1000 nm (for simultaneous imaging of GluSnFR3 + RCaMP2). The laser power coming through the objective was controlled with a Pockel’s cell and ranged from 10 to 40 mW for these experiments. Image acquisition was controlled through Scanimage software (Vidrio). Imaging was always performed in awake animals. Images (256 × 512 pixels at zoom values ranging from 8× to 12.1×, corresponding to ~64 × 128 μm to ~42 × 85 μm) were recorded at approximately 58.3 Hz in 5-min-on, 5-min-off intervals for the duration of the behavioral session to limit photo-damage to tissue. Imaging of a given field was performed in 5-day intervals, with three fields being selected for each animal, such that field 1 was imaged on sessions 1, 6, and 11, field 2 on sessions 2, 7, and 12, and field 3 on sessions 3, 8, and 13.

Near-simultaneous dual-plane somatic and dendritic imaging was performed using a commercial two-photon microscope (Ultima 2Pplus, Bruker) equipped with an optically corrected electrotunable lens (ETL), a 16×/0.8-NA objective (Nikon), and a Ti-Sa laser (Spectra-Physics) tuned to 1000 nm for RCaMP2 imaging. Laser power was controlled with a Pockel’s cell and ranged from 30 to 50 mW for these experiments. Image acquisition was controlled through Prairie View software (Bruker). Imaging was performed in awake animals. Images were captured at 512 × 512 pixels at 3× or 4× zoom, corresponding to ~458 × 458 μm and ~344 × 344 μm, respectively. Before time-series acquisition, a z-stack of the selected soma and its apical dendritic arbor was captured (1- to 2-μm increments; 30 frames per plane). Near-simultaneous dual-plane time series were then acquired at 15 Hz (per imaging plane), with one plane focused on the soma and the other plane focused on the apical dendritic arbor (~100 μm above the soma). Time series were continuously recorded for 10 to 20 min as mice were allowed to freely press the lever.

### Movement analysis

Movement analyses were performed as previously described (*9*, *23*). Briefly, lever displacement traces (voltage recordings from the force transducer) were down-sampled from 10 kHz to 1 kHz, then filtered using a four-pole 10-Hz low-pass Butterworth filter, after which the velocity of the lever was determined by smoothing the difference of consecutive points with a moving average window of 5 ms. The envelope of the lever velocity was then extracted using a Hilbert transform, and tentative movement bouts were defined by the envelope crossing a threshold of 4.9 mm per second. Each movement bout was extended by 75 ms on either side. Bouts separated by less than 500 ms were considered continuous. Movement start and end times were defined as the points at which the lever exceeded or fell below the thresholds defined by rest periods before and after the movement bouts. Thresholds were defined as the resting position plus the 99th percentile of the noise distribution, in turn defined as the difference between the Butterworth smoothed trace and the original trace. Individual movements were defined as the periods when the lever surpassed this threshold.

To compare the kinematic features of movements associated with spine activity, individual movements coincident with a given spines’ active periods (i.e., when the GluSnFR fluorescence trace is above threshold) were extracted for each spine. Windows of ~0.5 s before and 1.5 s after movement onset were used to equalize the duration of movement periods. Movements that overlapped after considering this window were excluded from consideration. The “stereotypy” of movements was calculated as the Pearson correlation coefficient between all such movements associated with a given target spine’s activity. The LMP was defined as the average of rewarded movements that started after cue onset from the late (*11*–*14*) learning sessions. Correlation with the LMP was defined as the average Pearson’s correlation coefficient between the LMP and all movements associated with a given target spine’s activity. Prolonged movements (lasting >3 s) typically corresponded to repeated movements in succession and were therefore excluded from this analysis.

### Image analysis

Images were analyzed as previously described (*9*). Briefly, lateral motion of imaging time series was first corrected using custom full-frame cross-correlation image alignment (*47*). Regions of interest (ROIs) were then manually drawn using custom MATLAB software. For dendritic spines, elliptical ROIs were drawn around the center of the spine head beyond the edge of detectable fluorescence above background, as previously reported (*9*). Apparent spines along the *z* axis of the dendritic shaft were included in analysis. Series of regularly spaced elliptical ROIs were also drawn along the length of the dendrite, with the center of each ellipse serving as the point along a poly-line being used to calculate dendritic distance between spines. All unique dendritic pixels falling within these elliptical ROIs were subsequently pooled for analysis of dendritic fluorescence (see the next section). A single large ROI was drawn in an empty region of the field to estimate background.

For display purposes in figures, images were manually cropped around dendrites of interest for visual clarity. Care was taken (using fluorescence traces as reference) to ensure that no structures belonging to the dendrite of interest were removed in the process.

### Fluorescence analysis

Fluorescence analysis was processed as previously described (*9*). Briefly, pixels within each ROI were averaged to create fluorescence time series for all imaging frames. The time-varying baseline (*F*_0_) of a fluorescence trace was estimated by smoothing inactive portions of the trace, using a previously described iterative procedure (*9*, *48*). The normalized Δ*F*/*F*_0_ trace was then calculated, where Δ*F* was found by subtracting the baseline trace from the raw trace, and *F*_0_ is the calculated time-varying baseline. Normalized Δ*F*/*F*_0_ traces were then denoised using the smooth function in Matlab with a 0.5-s window.

Activity events were detected based on previously described methods (*9*, *23*). Briefly, noise was estimated for each Δ*F*/*F*_0_ trace as the SD of negative fluorescence values mirrored about the origin. This noise estimate was then used to set two thresholds, one being 2× the noise to find active portions of the trace, and another being 1× the noise to define the baseline. Active portions of the trace were defined as when the 1-s LOESS-smoothed Δ*F*/*F*_0_ trace crossed the active threshold and extended backward to begin when the baseline threshold was crossed by the unsmoothed trace. For iGluSnFR2 traces, a lower limit for events was set at 0.2 to remove spurious event detection from noise in inactive spines, while a lower limit was not used for iGluSnFR3 due to its greater signal-to-noise ratio. Binarized traces with the value of 1 for active frames and 0 otherwise were then produced for each ROI. Such binarized traces were used for all co-activity analysis and event frequency calculations.

Structural analysis was performed a previously described (*9*). Briefly, average projection images of the entire motion-corrected time series were produced. To estimate spine size in each session, the summed fluorescence intensity of pixels with intensity values above background (the average pixel intensity across the designated background ROI) over a given spine ROI was divided by the average fluorescence intensity of the nearby region of dendrite for normalization. Such normalization should account for global changes in the expression level of the sensor or changes in optical conditions in the imaging field. Local dendritic fluorescence intensity was estimated by using the dendritic pixels (described above) within 20 μm of dendritic distance from the base of the spine. Normalization of the summed spine intensity by the mean intensity of the dendrite (intensity value per pixel) yields an estimate of the pixels in a given ROI that are above the calculated threshold based on background intensity. Spine areas were then calculated by accounting for pixel size based on the zoom value of a given image.

To confirm that structural readouts with iGluSnFR3 are robust and not biased by its glutamate responses, we repeated some control measurements that we performed for iGluSnFR2 in our previous report (*9*). For structural analysis with active periods removed, the same process described above was iteratively repeated for each spine with the exception that frames where a given spine was active (determined using the binary activity trace) were removed before generating average projection images. For structural comparisons between images captured at 925- and 810-nm excitation wavelengths, average projection images were first aligned to compensate between any shift between the two images. ROIs were then drawn using the 810-nm projection image as a reference, before the fluorescence was extracted as described above for both images using the same set of ROIs.

For RCaMP2 imaging, the pixels within dendritic ROIs were pooled and used to calculate Δ*F*/*F*_0_ traces of dendritic activity. Otherwise, dendritic activity was treated identically to spine activity described above, with the exception that an upper threshold of 3× the noise rather than 2× was used for detecting activity events.

### Evaluation of dendritic health

In our previous work (*9*), we designed the punctuated imaging schedule used in this study to minimize the phototoxic effects of dendritic imaging. As in this prior study, dendritic health was evaluated based on (i) the preservation of spine density, (ii) dendritic morphology, with any “blebbing” indicative of poor cellular health, and (iii) spine event frequency, with global decreases possibly indicating damage. We previously showed that these features did not change substantially from early to late sessions (i.e., the duration of the experiment).

For GluSnFR3-RCaMP2 coexpression experiments, we added additional requirements that RCaMP2 baseline fluorescence was preserved (without substantial bleaching within or across sessions) and at least one dendritic event was detected over both early and middle sessions (from which the activity was analyzed). After pilot experiments were conducted to establish appropriate virus ratios and imaging power, all imaged dendrites fulfilled these criteria.

### Spine structural classification

Average projections of time series from each session were registered with respect to the first imaging session for that field to allow for comparison across days. A duplicate set of these images was iteratively deconvolved using the “Interative Deconvolve” plugin for ImageJ as a guide for spine detection. We excluded spines that were too close to each other to be accurately separated for fluorescence trace extraction in the original two-photon image series projection. Any visible dendritic protrusion emanating from the dendrite was considered spines. Bright, punctate regions of at least 0.5 μm diameter overlapping with the dendrite in the imaging plane were also considered spines. Both assumptions were corroborated in our previous publication with electron microscopy reconstructions of imaged dendrites (*9*). Spines that appeared in the same location across sessions, or whose neck originated from the same dendritic region and was similar in appearance and approximate location, were considered the same spine. Spines were considered “eliminated” if they were present on the first imaging/training session and absent on any subsequent training period (either “middle” or “late” sessions). Structural assessment of elimination was corroborated through inspection of GluSnFR traces of the candidate spine. A spine was considered stable if it was observed in two consecutive sessions, and the features of the spine in the earlier session of the two were used to predict its stability. New spines that formed over the experimental period were not considered for either category.

Using the above approach, we previously showed that all spines identified through in vivo two-photon imaging were true synapses (as identified in correlated electron microscopy of the same dendrites), that a majority of true synapses were successfully identified, and that indicators of dendritic health (e.g., spine density) are preserved over the duration of the experiment (*9*).

Plasticity events of individual spines were assessed based on comparisons between sequential imaging sessions. Using the spine area measurements described above (see the “Fluorescence analysis” section), spine area changes over sessions were calculated as the ratio of spine area from the *n*th session to the (*n* − 1)th session. For analyses of spine area changes nearby elimination events, the *n*th session was defined as the session on which the elimination event was first observed, and the (*n* − 1)th session as the most recent prior imaging session of the same field.

For spine elimination and formation, plasticity events were again defined based on sequential imaging sessions of the same field. Formation events were carefully classified based on the clear absence of a spinous protrusion on one session followed by an equally clear protrusion on the subsequent session. Our previous work identified many unique features of new spines identified in this way, supporting their classification. New spines that appeared and were subsequently eliminated (i.e., transient new spines) were counted as formation events for Fig. 2F, but were not considered in analyses of eliminated spines. It is possible that a subset of spines imaged during the early session were transient spines, but given the relative stability of cortical synapses and the lower level of ongoing synaptogenesis before training commences we have previously observed, they likely represent a small minority (*23*, *49*). Spine elimination was defined using similar criteria to new spines in terms of assessing when they were present versus absent. In very rare cases, eliminated spines observed on the middle imaging session appeared to re-emerge on the late session. On the basis of the rarity of these events and the possibility that they are distinct events from typical elimination, we excluded these events from further consideration.

### Defining small stable spines

Small stable spines were defined based on the distributions of spine sizes (see the “Fluorescence analysis” section). To extract a subpopulation of stable spines that represented similar size to eliminated spines, we iteratively compared the lower *x*-percentile of stable spines against the full population of eliminated spines, ranging from the 10th percentile to the 90th percentile of spine sizes (fig. S4). We identified a range of percentiles over which the ratio between the subsampled spine population and the eliminated spine population was close to 1, and Wilcoxon rank-sum tests returned a statistically insignificant result. We used the 40th percentile of stable spine areas, since this was the value closest to a ratio of 1 while also being not significantly different than the eliminated spine population.

### Movement-related classification

Spines were classified as movement-related on each individual session, as previously described (*9*). Briefly, the dot product of binarized lever traces (movements versus nonmovements, as detailed above) and continuous Δ*F*/*F*_0_ traces was calculated for each spine. This value was then compared to the dot products when shuffling the movement periods 10,000 times. The dot products of each of the shuffled traces with Δ*F*/*F*_0_ traces were then compared to the values of the actual data. Actual values that were above the 97.5th percentile of the shuffled distribution were considered “movement-related.”

### Distance analysis

In all analyses regarding interspine distance, a given spine pair’s distance value corresponded to the dendritic distance (as determined from single-plane two-photon images) from the base of the target spine to the base of a given partner spine. Note that this dendritic distance differs from Euclidian distance (direct distance between two points in the plane) in that the curvature of dendrites was considered.

All distance values were binned in 5-μm increments to simplify visualization and statistical comparisons. Bins correspond to dBin*_n_* < *d* ≤ dBin_*n*+1_ such that the 2.5-μm bin represents interspine distance values from 0 to 5 μm, the 7.5-μm bin corresponds to 5 < *d* ≤ 10 μm, the 12.5-μm bin to 10 < *d* ≤ 15 μm, and so on.

### Event analysis

For the analysis of individual event amplitudes and decay kinetics (fig. S1), individual events were first identified using the binary activity trace for each spine. The peak of the event was defined using the signal.find_peaks function from the scipy python package in a 3-s window starting 1 s before and ending 2 s after event onset, with the minimum distance between peaks set to 0.5 s and a minimum peak height set to the median + SD of the full 3-s peri-event period being inspected. The amplitude of the maximum peak was taken. We then calculated the decay kinetics of the event by fitting an exponential decay function to the trace between the event peak and end of the window using the optimize.curve_fit function from the scipy python package. Events where fitting failed were excluded from further analysis. For display purposes, outliers were removed before histogram plotting (fig. S1), which was defined as more than 3× the interquartile range of the data.

### Clustering analysis

For analyses of the spatial clustering of eliminated spines with other classifications of spines (Fig. 2 and fig. S2), the dendritic environment surrounding each eliminated spine was analyzed on the session before elimination. To assess MRS density nearby eliminated spines, the number of MRSs within 10 μm (of dendritic distance) of an eliminated spine was normalized by the total dendritic distance considered. This value was typically 20 μm (considering 10 μm on either side of a given eliminated spine). In cases where a target eliminated spine was closer than 10 μm to the edge of an imaged dendrite, the normalization factor was adjusted to account for this, making the normalization factor (nf) 10 μm ≤ nf ≤ 20 μm. To estimate the chance level of local MRS density, eliminated spine labels were shuffled across all dendrites such that stable-versus-eliminated classifications were randomized. The calculation of nearby MRS density was then repeated based on the new environment of randomly selected spines. Intrinsic to this shuffling is that the overall numbers and spatial distributions of spines remain constant, with only the labels altered.

To assess whether eliminated spines are spatially clustered with other plasticity event, nearest-neighbor analysis was used. Here, spines were assigned labels according to whether they displayed a given plasticity event on the following (*n + 1*) session. Each eliminated spine was then assigned a distance value based on the nearest such plasticity event occurring on the same dendrite. To estimate chance, eliminated spine and stable spine labels were randomized across all dendrites, and the nearest plasticity events for each “pseudo” eliminate spine were calculated. This process was repeated 1000 times to assess significance (see “Statistical analysis” section).

### Co-activity analysis

Co-activity rates between all possible spine pairs on the same dendrite were calculated using binarized event traces (binarization process defined in the “Fluorescence analysis” section) for the entire imaging session, as previously described (*9*). All periods where activity events were present in both spines (i.e., both spines were above threshold) were considered co-active periods. A single co-activity event was defined as the entire duration that both spines were continuously co-active. The co-activity rates were calculated as the number of such co-active events per unit time (events per minute). Co-activity rates were normalized to the geometric mean of the activity frequencies of both spines. Since the geometric mean is highly correlated with the calculated co-activity rates, normalization by this value allows better comparison between the relative co-activity between spine pairs showing different overall frequencies. All co-activity rates were calculated across the entire trace (i.e., in both movement and nonmovement periods, as well as across all trial epochs, including intertrial intervals and cue periods).

### Co-activity environment analysis

For comparisons of the co-activity rates of eliminated spines to their nearby and distant environments (Fig. 3B), three groups were defined based on each eliminated spine: (i) the “eliminated spine” group, consisting of the mean co-activity between the target eliminated spine and its nearby spines; (ii) the “nearby environment” group, consisting of the mean co-activity rates between spines nearby (≤10 μm) the target eliminated spine but excluding any eliminated spines; and (iii) the “distant environment” group, consisting of the mean local co-activity rates between spines farther than 10 μm from the target eliminated spine. For the distant environment group, target spines were first defined based on their distance from the target eliminated spine. Their “local” co-activity was then defined by considering spine pairs within 10 μm of each such spine. Any spine labeled as part of the nearby environment group was excluded from consideration when summarizing the local co-activity of the distant environment group. In cases of multiple eliminated spines existing on the same dendrite, spines within 10 μm of nontarget eliminated spines were also excluded from inclusion in the distant environment group to minimize the overlap between the nearby environment and distant environment groups. For each eliminated spine, the normalized co-activity rates between all spine pairs in each group were averaged such that each eliminated spine was assigned one value for each of the three groups.

### Statistical analysis

For Fig. 1, *P* values were calculated as the fraction of shuffled samples (i.e., spine labels 1000 times) in which the measure was larger (Fig. 1D) or smaller (Fig. 1, E and F) than the actual value. These tests were therefore one-sided.

For all other figures, linear mixed-effects models were used to account for effects of nested data structure. Terms included in each model were hypothesis-based, but were ultimately chosen based on comparison of log-likelihood performance of each model. Models were fit using the MATLAB “fitlme” function.

For comparisons between eliminated and stable spines’ event rates, areas, MRS categorization probability, movement encoding parameters, or AP relationship, models were constructed as

Response∼Stability+(1∣animal)+(Stability−1∣Animal)

where Response corresponds to the above metrics being compared; Stability denotes the fixed effect term of spine stability, with categorical labels of “stable” or eliminated; (1∣animal) denotes a random effect of the intercept term grouped by animals; and (Stability − 1∣Animal) denotes a random effect of the slope term considered independently from the intercept term (i.e., no correlation between intercept and slope).

For additional comparisons of these same measures when assessing the potential influence of what session spines were eliminated on (figs. S3, S4, S9, and S10), models with additional terms were constructed as

$$\begin{array}{c} \text{Response}∼\text{Stability}+\text{Session}+\text{Stability}:\text{Session}\\ +\left(1|\text{Animal}\right)+\left(\text{Stability}-1|\text{Animal}\right)\\ +\left(\text{Session}-1|\text{Animal}\right)+\left(\text{Stability}:\text{Session}|\text{Animal}\right) \end{array}$$

where terms are the same as above, with the addition of “Session” denoting the fixed effect term of the imaging session in which elimination was detected, categorical labels of “early” or late (Session − 1|Animal) denoting a random effect of the slope term considered independent from the intercept term grouped by animal, and (Stability:Session|Animal) representing the random effect on slope and intercept terms of the interaction between spine stability and session, grouped by animal.

For comparisons of co-activity rates that vary as a function of interspine distance (Fig. 3 and fig. S5), models were constructed as

$$\begin{array}{c} \text{Coactivity}∼\text{Stability}+\text{Distance}+\text{Stability}:\text{Distance}\\ +\left(1|\text{animal}\right)+\left(\text{Stability}-1|\text{Animal}\right)\\ +\left(\text{Distance}|\text{Animal}\right)+\left(\text{Distance}:\text{Stability}|\text{Animal}\right) \end{array}$$

where Coactivity denotes the normalized co-activity for a given spine pair, Stability represents the fixed effect of stable or eliminated categorization, Stability:Distance represents the fixed-effect interaction term between spine stability and interspine distance, (1∣animal) represents the random effect of intercept grouped by animal, (Stability − 1∣Animal) represents the random effect on the slope term (independent of the intercept term) of spine stability, (Distance∣Animal) represents the random effect of slope and intercept terms of interspine distances, grouped by animal, and (Distance:Stability∣Animal) represents the random effect on the slope and intercept terms of the interaction between spine stability and interspine distance, grouped by animal.

Models re-examining the same comparisons but with small stable spines were identical to the original models, with the exception that the stable spine population was subsampled to only include small stable spines. Models comparing the co-activity curves of small and large spines across the stable and eliminated populations (fig. S6) were constructed as

$$\begin{array}{c} \text{Coactivity}∼\text{Stability}+\text{Distance}+\text{Stability}:\text{Distance}\\ +\text{Area}+\left(1|\text{animal}\right)+\left(\text{Stability}-1|\text{Animal}\right)\\ +\left(\text{Distances}|\text{Animal}\right)+\left(\text{Area}-1|\text{Animal}\right) \end{array}$$

where terms are the same as above, with the addition of Area representing the fixed effects of spine size and (Area − 1∣Animal) representing the random effect of slope grouped by animal.

Models re-examining the same comparisons, but with spines separated out by sessions (fig. S6), were constructed as

$$\begin{array}{c} \text{Coactivity}∼\text{Stability}+\text{Distance}+\text{Stability}:\text{Distance}\\ +\text{Session}+\left(1|\text{animal}\right)+\left(\text{Stability}-1|\text{Animal}\right)\\ +\left(\text{Distances}|\text{Animal}\right)+\left(\text{Session}-1|\text{Animal}\right) \end{array}$$

where the terms are the same as above, with the addition of Session representing the fixed effects of imaging sessions and (Session – 1*|*Animal) representing the random effect of slope grouped by animal.

Sample sizes (*n*) are as follows: For iGluSnFR2-only imaging experiments (Figs. 1 to 3), 27 mice in total were used, based on surgical outcome; of these 27 mice, 25 had at least one field that remained healthy for the three imaging sessions (see the “Evaluation of dendritic health” section); of these 25 mice, 24 showed at least one eliminated spine in the imaged fields; 3 imaging fields were attempted in each mouse, but only a subset were used, based on successfully relocating the field and the lasting health of the imaged field, yielding 45 total fields across the 24 mice (~1.67 fields per mouse); within these fields, 86 total healthy dendrites were successfully captured across all three imaging sessions (~1.9 dendrites per field), and of these 86 dendrites, 72 showed at least one elimination event and comprised the data used in this study; across these 72 dendrites, a total of 2014 unique spines were imaged (including stable, eliminated, and new spines); spines were classified as stable when they were present on both the session preceding and the session coincident with the first observation of an elimination event on the same dendrite, and were therefore classified over early-to-middle-session (E-to-M) and middle-to-late-session (M-to-L) pairings. It was thus possible that individual fields, dendrites, and spines are represented for only one session transition block (e.g., E-to-M), or represented twice, once for both E-to-M and M-to-L blocks. This resulted in a total stable spine count of 1368 (E-to-M) and 1318 (M-to-L) stable spines (2686 total, with 1049 being stable across all imaged sessions and coincident with spine elimination on both session transitions, and therefore being counted twice). Eliminated spine classification was subject to the same criteria, resulting in E-to-M elimination (131 spines) or M-to-L elimination (121 spines), totaling 252 observed elimination events. For co-activity analyses, spine pairs were included only when both spines displayed activity during the session (i.e., showed at least one GluSnFR event) and were present on the same dendritic branch. For iGluSnFR3-RCaMP2 coexpression experiments (Fig 4), seven total mice were used, from which 12 dendrites from eight fields fit the criteria for inclusion (stated above); 11 of the 12 imaged dendrites showed at least one elimination event and were considered for subsequent activity analysis; all 11 dendrites were active (i.e., showed at least one RCaMP2 event) on both early and middle sessions; on these 11 dendrites, 325 unique spines were imaged; stable spine counts were 290 (E-to-M) and 227 (M-to-L), totaling to 517; eliminated spine counts were 21 (E-to-M) and 25 (M-to-L), totaling to 46. For dual-plane imaging of RCaMP2, 15 dendrite-soma pairs across four neurons and two mice were used to assess dendrite-soma activity relationships (Fig. 4 and fig. S6). On these same neurons, 50 total “sister” dendrites (pairs of dendrites sharing the same parent neuron) were considered. For isosbestic imaging (fig. S8), 682 spines from 13 dendrites across four mice were used for spine volume comparisons between 810- and 925-nm wavelengths.
