# Supplementary material for: Local and global predictors of synapse elimination during motor learning
Source: Sci Adv. 2024 Mar 15;10(11):eadk0540. doi: 10.1126/sciadv.adk0540 (PMC10942101; doi:10.1126/sciadv.adk0540)
Supplement: Supplementary file 1 — Figs. S1 to S11 [file sciadv.adk0540_sm.pdf]

Science Advances
AAAS

Supplementary Materials for

Local and global predictors of synapse elimination during motor learning

Nathan G. Hedrick *et al.*

Corresponding author: Takaki Komiyama, tkomiyama@ucsd.edu

Sci. Adv. **10**, eadk0540 (2024)
DOI: 10.1126/sciadv.adk0540

This PDF file includes:

Figs. S1 to S11

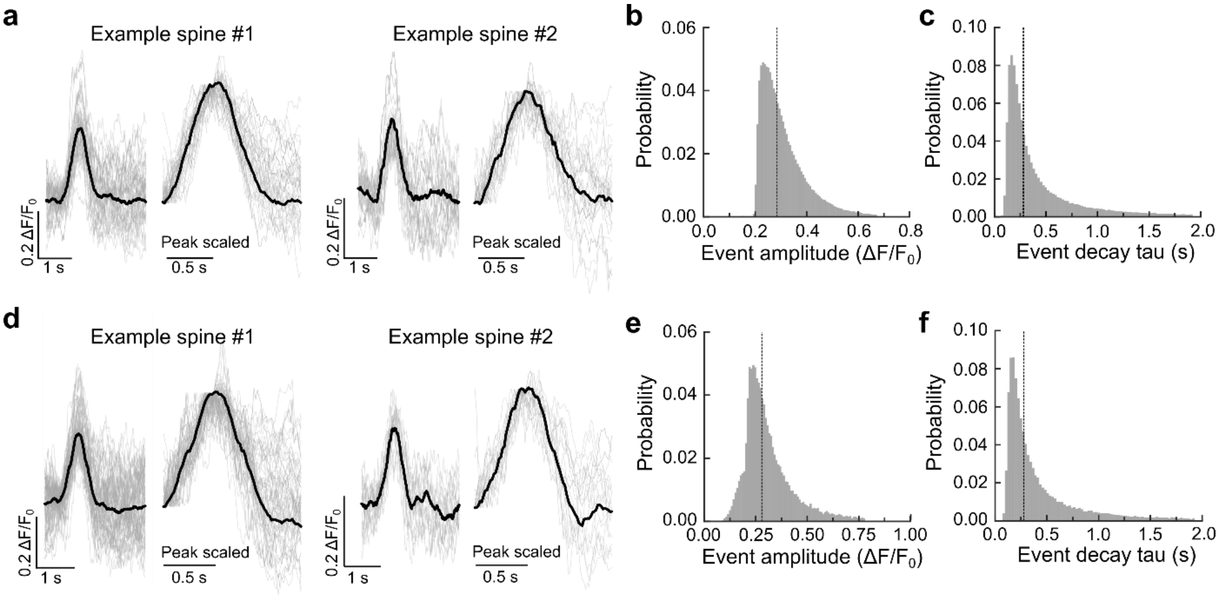

Figure S1. Properties of iGluSnFR2 and iGluSnFR3 events.

a) Example traces of individual (grey) and averaged (black) iGluSnFR2 events from two example spines. Right insets display peak scaled traces to illustrate variability in decay kinetics.

b) Histogram of the peak amplitude of all detected iGluSnFR2 events. Median = 0.285 dF/F. N = 327,945 events.

c) Histogram of the event decay kinetics of all detected iGluSnFR2 events fitted with exponential decay. The heavy tail of the distribution likely represents events consisting of bursts of presynaptic release resulting in prolonged synaptic activity. Median = 0.308 s. N = 327,945 events.

d) Same as **a**, but for iGluSnFR3 events.

e) Same as **b**, but for iGluSnFR3 events. Median = 0.283 dF/F. N = 62,139 events.

f) Same as **c**, but for iGluSnFR3 events. Median = 0.307s. N = 62,139 events.

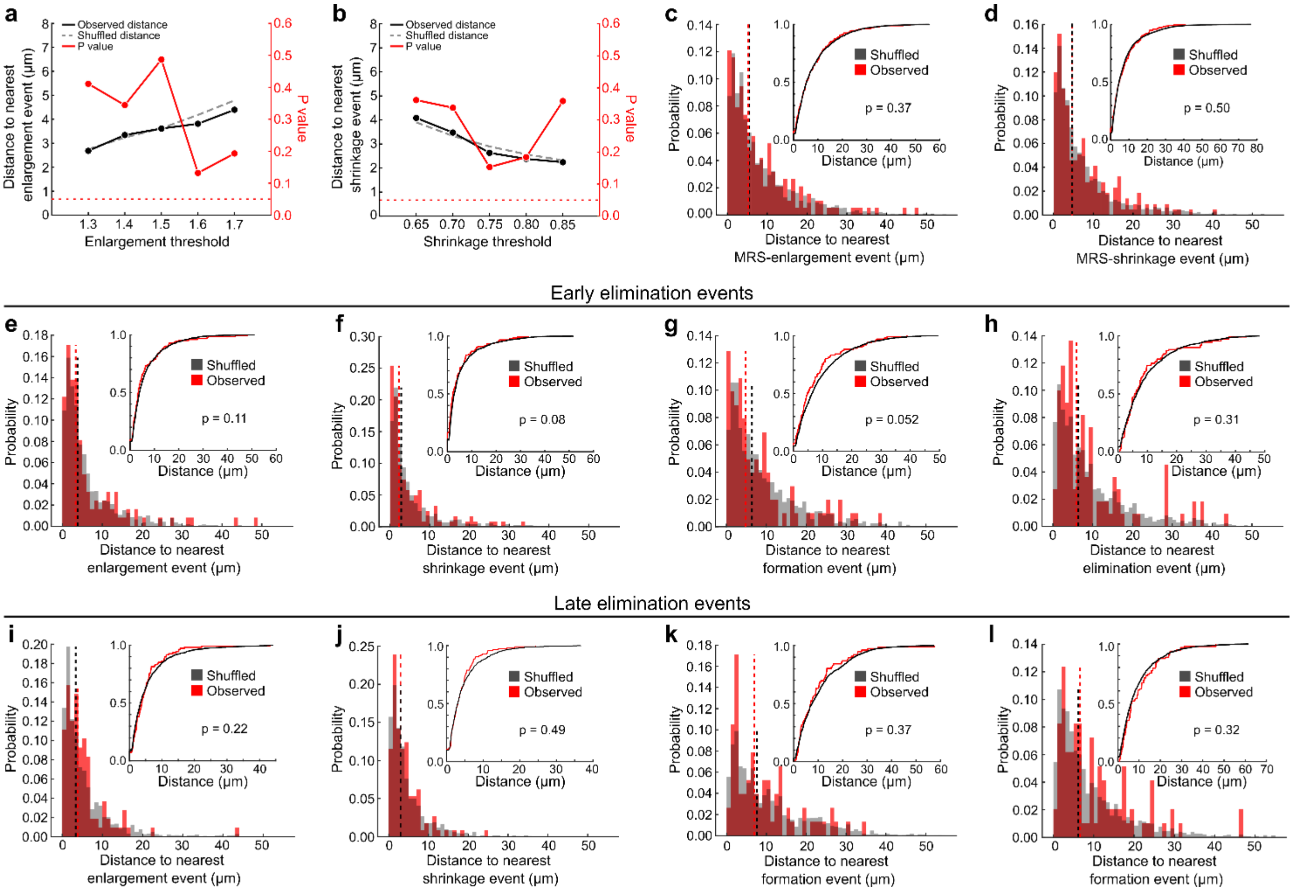

Fig. S2. Additional analyses examining the spatial clustering of spine elimination with other plasticity events

a) The lack of spatial clustering between spine elimination and enlargement events is not dependent on the enlargement threshold cutoff value. Nearest-neighbor distances between eliminated spines and enlargement events determined with varying cutoff values are plotted on left axis for both observed data (black) and shuffled data (grey dashed) with the corresponding p-values plotted on the right axis (red). There is no significant difference for any of the cutoff values.

b) The lack of spatial clustering between spine elimination and shrinkage events is not dependent on the shrinkage threshold cutoff value. Same as **A**, but for shrinkage events determined with varying cutoff values.

c) Histogram of nearest-neighbor distances between eliminated spines and enlargement events observed specifically in MRSs. All calculations are identical to **Fig 1e**, but using only MRSs. n = 24 mice / 252 eliminated spines / 313 enlarged MRSs.

d) Same as **c**, but for shrinkage events in MRSs. n = 24 mice / 252 eliminated spines / 457 shrunken MRSs.

e) Histogram of nearest-neighbor distances between eliminated spines and enlargement events that occurred between early and middle sessions. All calculations are identical **to Fig 1e**, but

using only events occurring between early and middle sessions. N = 24 mice / 131 eliminated spines / 339 enlarged spines.

f) Same as **e**, but for shrinkage events occurring between early and middle sessions. N = 24 mice / 131 eliminated spines / 449 shrunken spines.

g) Same as **e**, but for formation events occurring between early and middle sessions. N = 24 mice / 131 eliminated spines / 105 new spines.

h) Same as **e**, but for other elimination events occurring between early and middle sessions. N = 24 / 131 eliminated spines.

i) Histogram of nearest-neighbor distances between eliminated spines and enlargement events that occurred between middle and late sessions. N = 24 mice / 121 eliminated spines, 354 enlarged spines.

j) Same as **i**, but for shrinkage events occurring between middle and late sessions. N = 24 mice / 121 eliminated spines, 474 shrunken spines.

k) Same as **i**, but for spine formation events occurring between middle and late sessions. N = 24 mice / 121 eliminated spines, 74 new spines.

l) Same as **i**, but for other elimination events occurring between middle and late sessions. N = 24 mice / 121 eliminated spines.

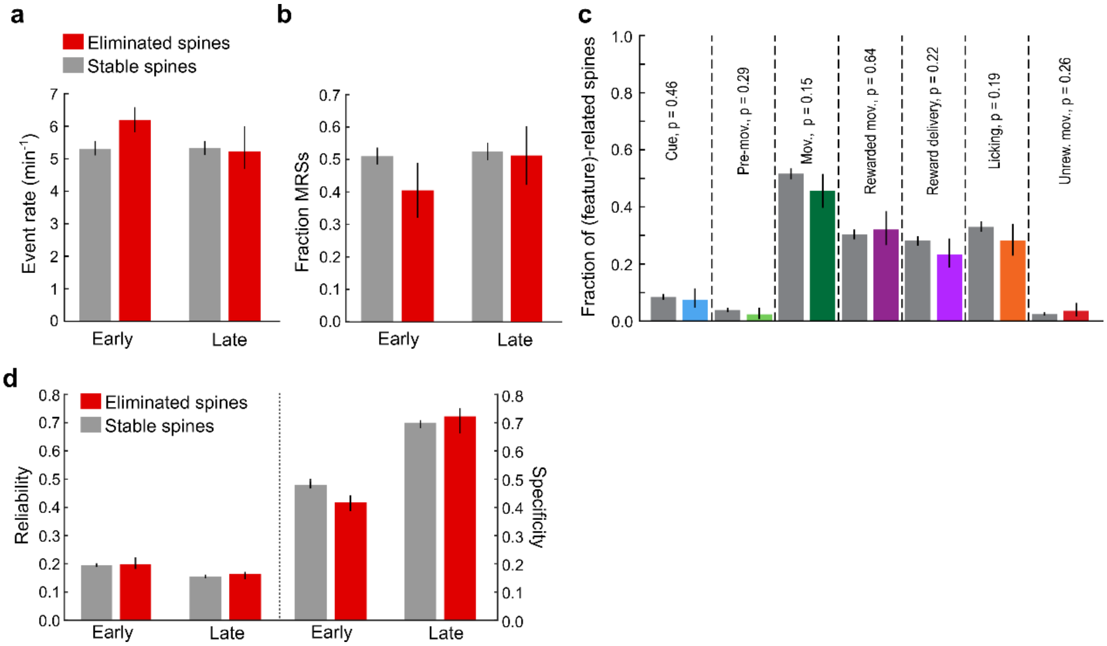

Fig. S3. Additional analyses of spine activity and task engagement

a) Event rates between stable (grey) and eliminated (red) spines separated by session. There is no effect of session on the event rates of eliminated or stable spines (Mixed-effects model, fixed effect of spine stability: p = 0.041; fixed effect of session: p = 0.932; fixed effect of spine stability × session interaction p = 0.268). n = 24 mice / 1368 early stable spines / 131 early eliminated spines / 1318 late stable spines / 121 late eliminated spines.

b) Fraction of stable (grey) and eliminated (red) spines that are classified as MRSs separated by session. There is no effect of session (Mixed-effects model, fixed effect of spine stability: p = 0.091; fixed effect of session: p = 0.737; fixed effect of spine stability × session interaction: p = 0.224). n = 24 mice / 1368 early stable spines / 131 early eliminated spines / 1318 late stable spines / 121 late eliminated spines.

c) Eliminated spines show comparable probability of being statistically related to different task features. Classification was performed similarly to MRS classification, but using periods corresponding to cue periods, pre-movement periods (100ms before movement onset), rewarded movement periods, reward delivery periods, licking bouts, and unrewarded movement periods. Grey bars correspond to stable spines, colored bars correspond to eliminated spines in the different classification categories. Bars represent mean, error bars represent 95% confidence intervals. Statistics reflect mixed-effects models accounting for grouping by animal.

d) Movement reliability (left) and movement specificity (right) of stable (grey) and eliminated (red) spines separated by session. Movement reliability decreases in later sessions for both eliminated and stable spines (Mixed-effects model, fixed effect of spine stability: p = 0.874; fixed effect of session: p = 2.34×10^{-14}; fixed effect of spine stability × session interaction: p = 0.953). Conversely, movement specificity increases in later sessions for both eliminated and stable spines (Mixed-effects model, fixed effect of spine stability: p = 0.014, fixed effect

of session: $p = 9.03 \times 10^{-10}$; fixed effect of spine stability × session interaction: $p = 0.137$). n = 24 mice / 1368 early stable spines / 131 early eliminated spines / 1318 late stable spines / 121 late eliminated spines.

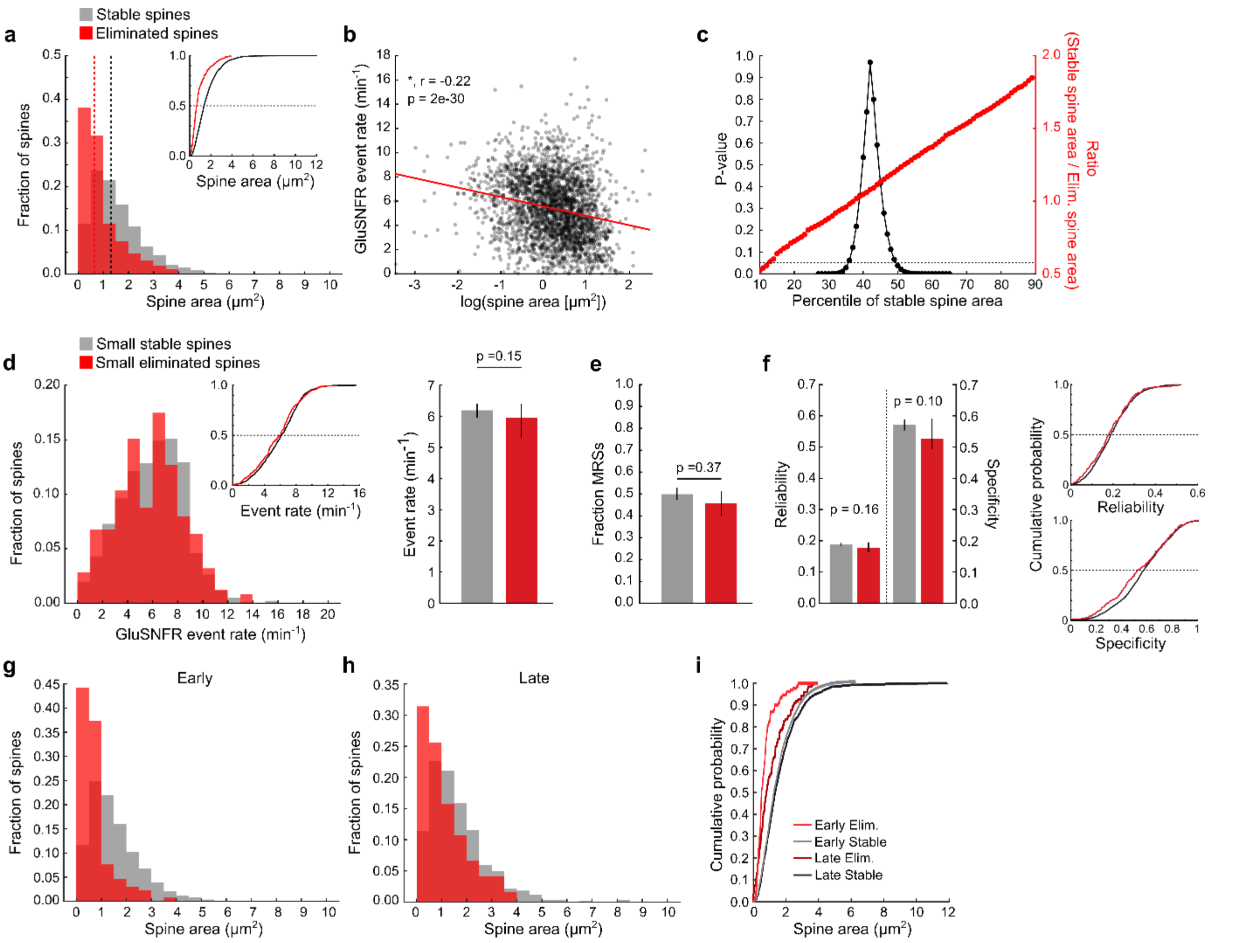

Fig. S4. The small sizes of eliminated spines likely account for their higher frequency and the lower movement specificity of their activity

a) Eliminated spines display relatively small areas. Histograms of spine areas for eliminated spines (red) and session-matched stable spines (gray). Dashed lines: medians of color-coded data. Inset: cumulative probability distributions of eliminated (red) and stable (black) spine areas. Black dashed line indicates 50th percentile. p = 3e-26, mixed-effects model.

b) Spine area significantly negatively correlates with iGluSnFR2 event frequency. Rates of iGluSnFR2 events in individual spines plotted as a function of spine area. Spine areas are log-transformed to account for the non-Gaussian distributions (a). Statistics represent Pearson's correlation coefficient between event rates and log-transformed spine area. Data points represent individual spines.

c) Statistics for the selection of "small" stable spines. The p-values of Wilcoxon rank-sum tests (left) between the putative small stable population and the entire eliminated spine population are plotted against the percentile used as a cutoff of "small" vs. "large". The ratios between the medians of the putative "small" population and the eliminated spine population are plotted on the right y-axis (red). We used the 40th percentile as the cutoff, by which the areas of small stable spines are statistically indistinguishable from those of eliminated spines. This yielded 1074 "small" stable spines out of a total of 2686 total stable spines.

d) Higher event rates observed in eliminated spines are likely related to their small size. Left, histograms of GluSnFR2 event rates for eliminated spines (red) and small stable spines. Inset, cumulative probability distributions of eliminated (red) and small stable (black) spines. Right, bar plot summarizing event rates between stable (gray) and eliminated (red) spines. Median ±bootstrapped 95% confidence intervals for this and all other bar graphs. $p = 0.15$, mixed-effects model. $n = 24$ mice / 1074 small stable spines / 252 eliminated spines.

e) Eliminated spines are equally likely to be categorized as MRSs when compared to small stable spines ($p = 0.37$, mixed-effects model). Bar summary of the mean fraction of spines meeting the criterion for categorization as MRSs. Rank-sum test.

f) Movement specificity differences between stable and eliminated spines are likely a product of their small sizes (**Fig. 2**). Left, bar summaries of the movement reliability and movement specificity shown for small stable spines (gray) and eliminated spines (red) (reliability: $p = 0.16$, mixed-effects model; specificity: $p = 0.10$, mixed-effects model). Right, cumulative probability distributions for the data summarized at left, for small stable (black) and eliminated (red) spines.

g) Same as **a**, but displaying spines eliminated or stable between early and middle sessions.

h) Same as **a**, but displaying spines eliminated or stable between middle and late sessions.

i) Cumulative probability distributions of early eliminated (light red), late eliminated (dark red), early stable (grey), and late stable (black) spine areas. Eliminated spines are smaller than stable spines, and there is no effect of session (Mixed-effects model, fixed effect of spine stability: $p = 2.87 \times 10^{-7}$, fixed effect of session: $p = 0.149$, fixed effect of spine stability × session interaction: $p = 0.413$). $n = 24$ mice / 1368 early stable spines / 131 early eliminated spines / 1318 late stable spines / 121 late eliminated spines.

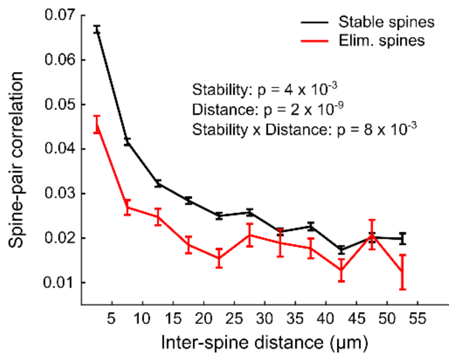

Fig. S5. Differences in functional clustering between stable and eliminated spines are robust to the metric of spine pair co-activity

Same as **Fig. 3a**, but using Pearson's correlation coefficients. Statistics: mixed-effects model. Fixed effect of distance: p = 2e-9; fixed effect of spine stability: p = 4e-3; fixed effects of stability × distance interaction: p = 8e-3. n = 24 mice / 2686 total stable spines / 252 total eliminated spines / 92967 stable spine pairings / 7756 eliminated spine pairings.

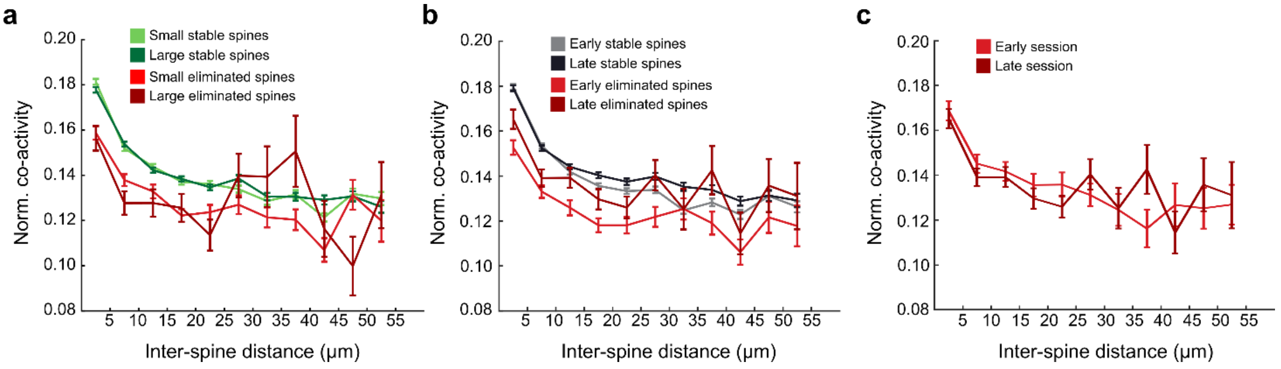

Fig. S6. The reduced functional clustering observed in eliminated spines is not due to their small size nor is it influenced by session.

a) Normalized pairwise co-activity rates for spines on the same dendrites as a function of inter-spine distance (distances binned in 5µm intervals). Separating stable (green shades) and eliminated (red shades) spines into small and large categories reveals no systematic differences in spine co-activity curves due to spine size. Statistics: mixed effects model. Overall, pairwise co-activity rates decay as a function of distance between spine pairs (fixed effect of distance, p = 1e-7) and are consistently lower for eliminated spines (fixed effect of stability, p = 0.01). The effect of spine stability on co-activity shows a significant interaction with distance (fixed interaction term: distance x stability, p = 0.01) such that the difference is smaller at larger distances. Spine size does not systematically affect co-activity rates (fixed effect of area, p = 0.34). n = 24 mice / 2686 total stable spines / 252 total eliminated spines / 39841 small stable spine pairings / 53126 large stable spine pairings / 6061 small eliminated spine pairings / 39841 large eliminated spine pairings.

b) Same as in **a**, but separating eliminated (red shades) and stable (grey shades) spines by training session. Overall, training session does not systematically affect co-activity rates (Mixed-effects model; fixed effect of distance: p =0.553, fixed effect of spine stability: p = 6.62×10^{-5}, fixed effect of session: p = 0.095, fixed effect of distance × spine stability interaction: p = 0.039). N = 24 mice / 2686 total stable spines / 252 total eliminated spines / 48081 early stable spine pairings / 44886 late stable spine pairings / 4471 early eliminated spine pairings / 3285 late eliminated spine pairings.

c) Same as in **a**, but displaying the pairwise co-activity for late eliminated spines during early and middle sessions separately. Overall, the coactivity of late eliminated spines does not change over the course of training prior to their elimination (Mixed-effects model; fixed effect of distance: p = 1.99×10^{-9}, fixed effect of session: p = 0.124, fixed effect of distance x session interaction: p = 0.452). N = 24 mice / 121 late eliminated spines / 2518 early session spine pairings / 3285 middle session spine pairings.

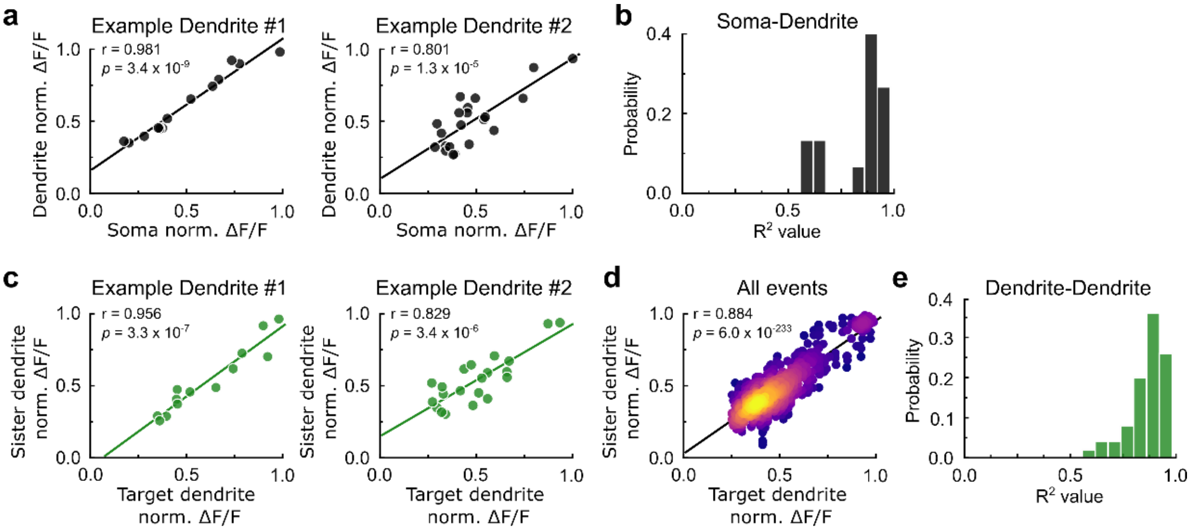

Fig. S7. Analysis of the Ca^{2+} event-coupling between postsynaptic compartments

a) Individual examples of dendrite-soma pairs representing the upper (left) and lower (right) bounds in the coupling of dendritic and somatic Ca^{2+} events observed. The amplitude of normalized Ca2+ events in dendrites is plotted against the same value calculated at the parent soma. Data points represent individual dendritic events. Statistics correspond to Pearson's correlation coefficient.

b) Distribution of correlations between dendritic and somatic normalized Ca^{2+} amplitude across all dendrite-soma pairs. Histogram of amplitude correlation R^2 values for individual dendrite-soma pairs. n = 2 mice / 4 neurons / 15 dendrite-soma pairs.

c) Individual examples of sister dendrite pairs (different branches of the same dendrite) representing the upper (left) and lower (right) bounds in the coupling of sister dendrite Ca^{2+} events. The amplitude of normalized Ca^{2+} events in dendrites is plotted against the same value calculated at a sister dendrite. Data points represent individual dendritic events. Statistics correspond to Pearson's correlation coefficient.

d) Ca^{2+} events in sister dendrites are tightly coupled in L2/3 neurons in M1. The amplitude of normalized Ca^{2+} events in dendrites is plotted against the same value calculated at the sister dendrite. Data points represent individual dendritic events pooled across sister dendrite pairing. Points are color-coded using a kernel density function to disambiguate crowded data points. Statistics correspond to Pearson's correlation coefficient. n = 2 mice / 4 neurons/ 50 sister dendrite pairs/ 701 total paired events.

e) Distribution of correlations between dendritic and sister dendritic normalized Ca^{2+} amplitude across all sister dendrite pairs. Histogram of amplitude correlation R^2 values for individual sister-dendrite pairs. n = 2 mice / 4 neurons / 50 sister dendrite pairs.

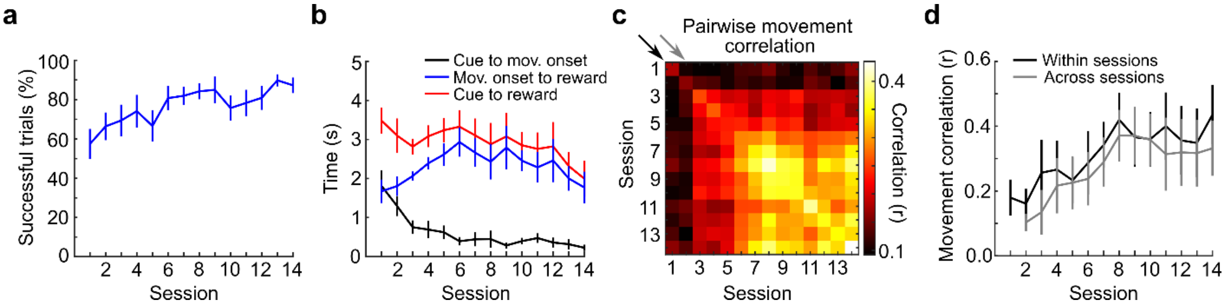

Fig. S8. Behavior data for iGluSnFR3-RCaMP2 co-expressing mice

- **a)** The percentage of trials resulting in reward increases over learning ($p = 2.53 \times 10^{-5}$; Pearson's correlation). n = 7 mice.
- **b)** The reaction time (black) and the time from cue to reward (red) decrease over learning ($p = 1.14 \times 10^{-8}$ for reaction time; $p = 1.50 \times 10^{-10}$ for cue to reward; Pearson's correlation). Time from movement onset to reward (blue) initially increases and then begins to decrease (p = 0.66; Pearson's correlation). n = 7 mice.
- **c)** Correlogram of lever trajectory correlation within and across sessions (n = 7 mice). Each box corresponds to the median pairwise correlation coefficient of rewarded movement trajectories over a 3s window.
- **d)** The mean lever trajectory correlation increases both within (black; center diagonal from **c**; p = 0.0015; Pearson's correlation) and across (gray; +1 diagonal from **c**; p = 0.0038; Pearson's correlation) learning sessions. N = 7 mice.

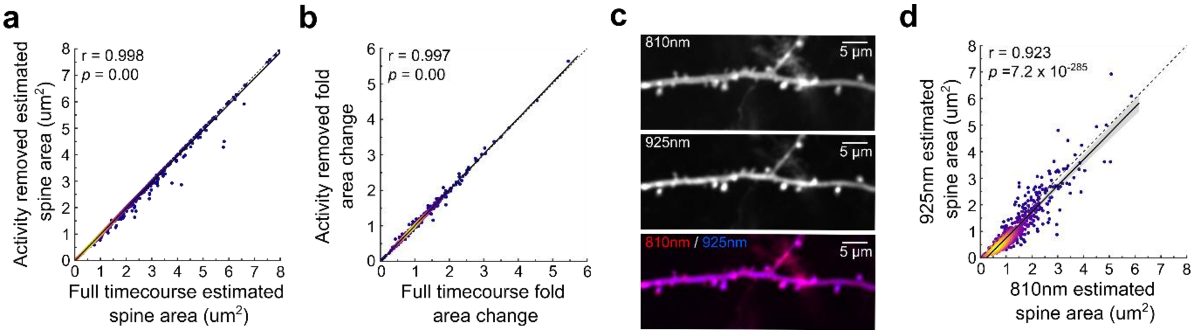

Fig. S9. *in vivo* structural analysis using iGluSnFR3 is not affected by spine activity.

a) Evaluation of the effect of removing active periods for each spine from image projections prior to calculation of spine area on the estimation of spine area. 'Activity-removed' area estimates strongly and significantly correlate with spine area estimates calculated from full-length time series (Pearson's correlation: r = 0.998, p = 0.00), indicating ongoing spine activity does not alter spine area estimation. Unity line shown with black dashed line and linear fit shown with black solid line. N = 7 mice / 1575 spines.

b) Evaluation of the effect of removing active periods from projections prior to calculation of spine area on the estimation of spine area change. 'Activity-removed' area change estimates strongly and significantly correlate with spine area changes calculated from full-length time series (Pearson's correlation: r = 0.997, p = 0.00), indicating ongoing spine activity does not affect the estimation of spine area changes. N = 7 mice / 782 spines.

c) Example images of the same dendrite imaged at 810nm and 925nm excitation wavelengths. 810nm is near the isosbestic point of iGluSnFR3 such that iGluSnFR3 fluorescence is independent of glutamate concentration. All spines visible at 810nm remained visible at 925nm, indicating spine activity does not impede the detection of dendritic spines.

d) Comparison of estimated areas of the spines imaged at 810nm and 925nm wavelengths. 810nm area estimates strongly and significantly correlate with 925nm area estimates (Pearson's correlation: r = 0.923, $p = 7.2 \times 10^{-285}$), indicating ongoing spine activity does not affect the estimation of spine area. N = 4 mice / 682 spines.

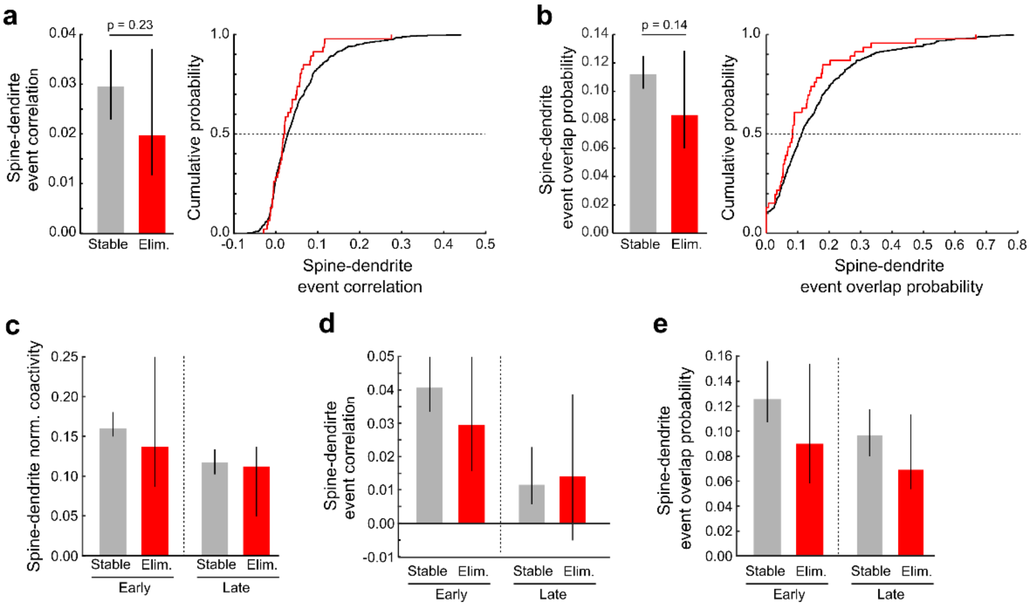

Fig. S10. Analysis of other metrics of coincidence of spines' activity with dendritic calcium events

a) Same as **Fig. 5c**, but using correlation coefficients between spine and dendrite events. Left, bar summaries representing median correlation values. Error bars represent bootstrapped 95% confidence intervals. Right, cumulative probability plot of the data at left. $p = 0.23$, mixed-effects model.

b) Same as **Fig. 5c**, but using the overlap probability of spine and dendrite events. Overlap probability is defined as the fraction of spine event periods that overlap with dendritic calcium events. Left, bar summaries representing median overlap probabilities. Error bars represent bootstrapped 95% confidence intervals. Right, cumulative probability plot of the data at left. $p = 0.14$, mixed-effects model.

c) Same as **Fig. 5c**, but separating spines by session. Bar summaries represent median normalized coactivity values. Error bars represent bootstrapped 95% confidence intervals. Mixed-effects model; fixed effect of spine stability: $p = 0.55$, fixed effect of session: $p = 1.72 \times 10^{-10}$, fixed effect of spine stability × session interaction: $p = 0.845$. N = 7 mice / 290 early stable spines / 23 early eliminated spines / 227 late stable spines / 25 late eliminated spines.

d) Same as **a**, but separating spines by session. Bar summaries represent median correlation values. Error bars represent bootstrapped 95% confidence intervals. Mixed-effects model: fixed effect of spine stability: $p = 0.379$, fixed effect of session: $p = 6.75 \times 10^{-7}$, fixed effect of spine stability × session interaction: $p = 0.621$). N = 7 mice / 290 early stable spines / 23 early eliminated spines / 227 late stable spines / 25 late eliminated spines.

e) Same as **b**, but separating spines by session. Bar summaries represent median overlap probabilities. Error bars represent bootstrapped 95% confidence intervals. Mixed-effects model: fixed effect of spine stability: $p = 0.911$, fixed effect of session: $p = 5.31 \times 10^{-12}$,

fixed effect of spine stability x session: $p = 0.841$. N = 7 mice / 290 early stable spines / 23 early eliminated spines / 227 late stable spines / 25 late eliminated spines.

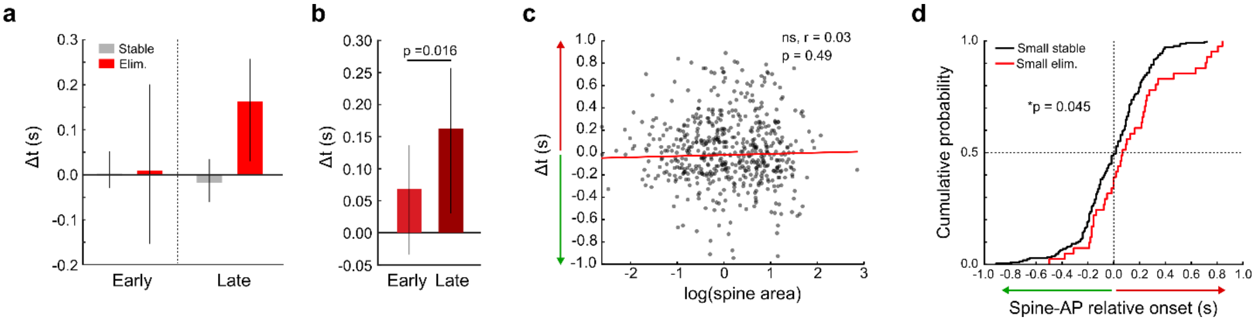

Fig. S11. Relative timing of spine activity vs. dendritic calcium events does not depend on spine size

a) Same as **Fig 5d**, but with spines separated by session. Bar summaries represent the median Δt values. Error bars represent bootstrapped 95% confidence intervals. Mixed-effects model, fixed effect of spine stability: $p = 0.578$, fixed effect of session: $p = 0.752$, fixed effect of spine stability × session interaction: $p = 0.109$. N = 7 mice / 281 early stable spines / 20 early eliminated spines / 177 late stable spines / 21 late eliminated spines.

b) Same as **a**, but displaying the relative timing of spine activity vs. dendritic calcium for late eliminated spines during early and middle sessions separately. Late eliminated spines display a relatively delayed event onset during early sessions, but this significantly increases during later sessions (Mixed effect model: fixed effect of session: $p = 0.016$). N = 7 mice / 21 eliminated spines.

c) Relative timing of spine activity vs. dendritic calcium events does not significantly correlate with spine size. Δt values, as in **Fig. 5d-e**, are plotted against log-transformed spine areas (to account for the non-Gaussian distribution of areas, see **Fig. S3f**). Green and red arrows on the y-axis represent the delineation between spines whose activity occurs, on median, prior to dendritic calcium event onset (negative values, green arrow) vs. spines whose activity occurs after dendritic calcium event onset (positive values, red arrow). Statistics represent Pearson's correlation coefficient.

d) Eliminated spines' delayed activity relative to dendritic calcium events is unlikely to be due to their small size. Same as **Fig. 5d**, but considering only small stable spines. $p = 0.045$, mixed-effects model.
